# Uncovering the mesendoderm gene regulatory network through multi-omic data integration

**DOI:** 10.1016/j.celrep.2022.110364

**Published:** 2022-02-15

**Authors:** Camden Jansen, Kitt D. Paraiso, Jeff J. Zhou, Ira L. Blitz, Margaret B. Fish, Rebekah M. Charney, Jin Sun Cho, Yuuri Yasuoka, Norihiro Sudou, Ann Rose Bright, Marcin Wlizla, Gert Jan C. Veenstra, Masanori Taira, Aaron M. Zorn, Ali Mortazavi, Ken W.Y. Cho

**Affiliations:** 1 Department of Developmental and Cell Biology, University of California, Irvine, CA, USA; 2 Center for Complex Biological Systems, University of California, Irvine, CA, USA; 3 Laboratory for Comprehensive Genomic Analysis, RIKEN Center for Integrative Medical Sciences, Yokohama, Japan; 4 Department of Anatomy, School of Medicine, Toho University, Tokyo, Japan; 5 Department of Molecular Developmental Biology, Radboud University, Nijmegen, the Netherlands; 6 Division of Developmental Biology, Department of Pediatrics, Cincinnati Children’s Hospital Medical Center, University of Cincinnati College of Medicine, Cincinnati, OH, USA; 7 Department of Biological Sciences, Chuo University, Tokyo, Japan; 8 These authors contributed equally; 9 Senior author; 10 Lead contact

## Abstract

Mesendodermal specification is one of the earliest events in embryogenesis, where cells first acquire distinct identities. Cell differentiation is a highly regulated process that involves the function of numerous transcription factors (TFs) and signaling molecules, which can be described with gene regulatory networks (GRNs). Cell differentiation GRNs are difficult to build because existing mechanistic methods are low throughput, and high-throughput methods tend to be non-mechanistic. Additionally, integrating highly dimensional data composed of more than two data types is challenging. Here, we use linked self-organizing maps to combine chromatin immunoprecipitation sequencing (ChIP-seq)/ATAC-seq with temporal, spatial, and perturbation RNA sequencing (RNA-seq) data from *Xenopus tropicalis* mesendoderm development to build a high-resolution genome scale mechanistic GRN. We recover both known and previously unsuspected TF-DNA/TF-TF interactions validated through reporter assays. Our analysis provides insights into transcriptional regulation of early cell fate decisions and provides a general approach to building GRNs using highly dimensional multi-omic datasets.

## In brief

Building mechanistic gene regulatory networks (GRNs) from highly dimensional genomic datasets to recapitulate cellular differentiation remains a difficult problem in developmental biology. Jansen et al. use linked self-organizing maps to combine ChIP-seq/ATAC-seq with temporal, spatial, and perturbation RNA-seq data from *Xenopus tropicalis* mesendoderm development to build a high-resolution genome-scale mechanistic GRN.

## Graphical Abstract

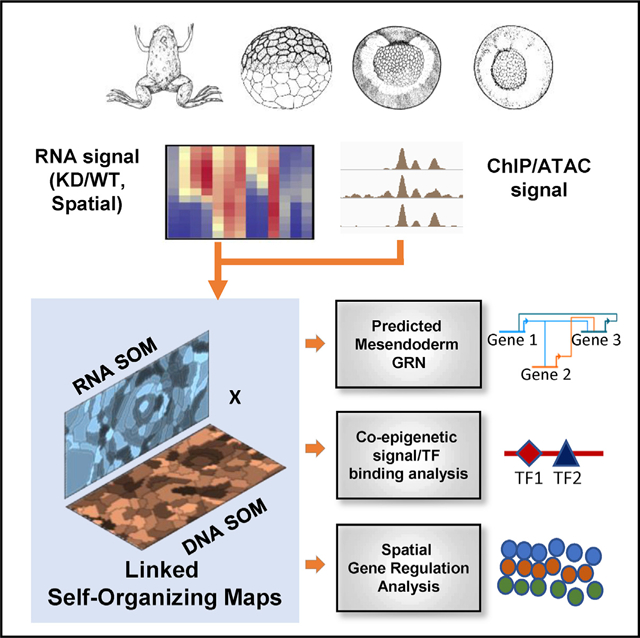

## INTRODUCTION

Understanding the transcriptional control of cellular differentiation programs is fundamentally important in biology and regenerative medicine. Simple linear pathways of gene regulation are insufficient to explain complex biological phenomena. This is because genes function in complex networks, and the emergent properties of these networks ultimately generate biological outcomes (Levine and Davidson, 2005; Davidson, 2010). Thus, identifying network structure is a necessary step toward comprehending the causes of cellular states and behaviors in embryogenesis, in adults, and in disease.

Efforts were made to compile the available molecular data into gene regulatory networks (GRNs) describing mesendoderm (ME) development in *Xenopus* (Loose and Patient, 2004; Koide et al., 2005). Arguably, these developmental events represent most influential developmental periods in metazoan organisms, leading to large-scale morphogenetic changes and body axis formation. Recently, a highly curated interactome map was generated based on over 200 publications (Charney et al., 2017a), which represents the most thoroughly examined ME GRN in any chordate. It revealed that germ layer specifications are controlled by a set of transcription factors (TFs) acting in a complex network. Additionally, previous work has suggested that critical aspects of the ME GRN are conserved in all vertebrates (Zorn and Wells, 2009). Therefore, a highly robust *Xenopus* GRN is likely to inform conserved paradigms in human development and strategies to direct human cell differentiation.

Although the current GRN has revealed critical principles governing early embryonic development (Charney et al., 2017a; Paraiso et al., 2020), it is far from complete. This is in part because, in the past, network connections were not fully embedded within the larger regulatory architecture nor included temporal and spatial data. Thus, the ME GRN is likely to miss many important interactions. An alternative approach is to generate a GRN based on a combination of computational methods with extensive perturbation analysis. Availability of large-scale genomics datasets in recent years allows us to test the utility of such an approach. However, one difficulty is the potential to produce numerous putative interactions that may contain false positives. This concern is supported by chromatin immunoprecipitation sequencing (ChIP-seq) analyses, which often uncover tens of thousands of TF-bound sites, but only a fraction of such sites directly affect gene expression (Li et al., 2008; Kvon et al., 2012). Therefore, it would be valuable to develop an approach that embraces the scale of the genomic data, while minimizing false positives. Although other methods have been developed to solve this problem through refinement of peak calling of ChIP-seq datasets (Bardet et al., 2013), we hypothesized that integration of genetic perturbation data types into the analysis of chromatin datasets would allow for a more informed identification of functional binding sites.

Given the current accumulation of large genomic datasets, computational GRN inference has become a popular field of research. One common way that these methods operate is using co-expression matrices to build “influential” GRNs (reviewed in Delgado and Gómez-Vela, 2019), which rely on correlations between genes rather than direct mechanistic regulation between TFs and target genes. The vast majority of GRN inference studies using high-throughput data build networks of this type. For the current work, we wished to build a “mechanistic” GRN, so we sought to find direct connections that engage *cis*-regulatory regions. This is extremely difficult using only one type of data (reviewed in Hu et al., 2020). Some recent predictive algorithms use multi-omic data to build lists of putative functional enhancers (Sethi et al., 2020; Xiang et al., 2020), but they do not incorporate TF binding data to determine whether the TF can directly regulate these enhancers. Others focus on integrating single-cell data types, e.g., Seurat/Cicero (Stuart et al., 2019), but they can create “influential” GRNs only from gene correlation matrices. Here, we describe the use of widely available bulk data for constructing mechanistic GRNs.

We adapted our linked self-organizing map (SOM) method (Jansen et al., 2019) to the multiple data types available for ME development: ChIP-seq, ATAC-seq, and RNA-seq of wild-type (temporal and spatial) and perturbation conditions. SOMs (reviewed in Kohonen, 2001) are a type of unsupervised neural network that train on a set of data to generate a low-dimensional representation. Previous works have successfully used the SOM’s remarkable ability to generate robust clusters (Kiang and Kumar, 2001) by incorporating them into the analysis of highly dimensional genomic data. For example, SOMs have identified complex relationships between genes and genomic regions in multiple cell types in human and mouse (The ENCODE Project Consortium, 2012; Mortazavi et al., 2013; Cheng et al., 2014; Yue et al., 2014; Partridge et al., 2020). The linked SOM method combines the clustering of multiple SOMs, each built with a different type of data (e.g., ChIP-seq and RNA-seq), into one analysis.

Here, to apply the linked SOM method, we generated a clustering of genes by training a SOM on 95 transcriptomic RNA-seq datasets to capture gene expression profiles that co-vary across different experimental conditions. Similarly, a clustering of genome regions was generated by training a SOM over 63 ChIP/ATAC-seq (Assay for Transposase-Accessible Chromatin using sequencing) experiments. Next, we combined the RNA and DNA clusterings by associating the individual genomic regions within the DNA clusters to the closest gene. This generated a multi-clustering, in which each cluster contained genome region-gene pairs that had a similar DNA (in the genome regions) and RNA signal (in the genes). These linked metaclusters (LMs) are extremely similarly regulated, so motif analysis on the genome regions, even after strict enrichment filtering, is more successful than scanning the whole genome alone (Jansen et al., 2019), and thus identified many new functional TF-DNA interactions. These inferred interactions were validated using reporter gene assays, supporting that the linked SOM approach is a valuable method to build mechanistic GRNs. This method was also applied to the RNA-seq data from spatial dissections to find TF-gene pairs with unique spatial properties during ME development. This analysis found six TFs, which based on their gene expression profiles and the expression of their predicted targets, should be included in the core network. By extending the linked SOM method for *Xenopus* ME development, we have developed a useful approach to find TFs important for germ layer specification.

## RESULTS

### Reconciling the known biology with evidence from high-throughput data

**Figure 1.**
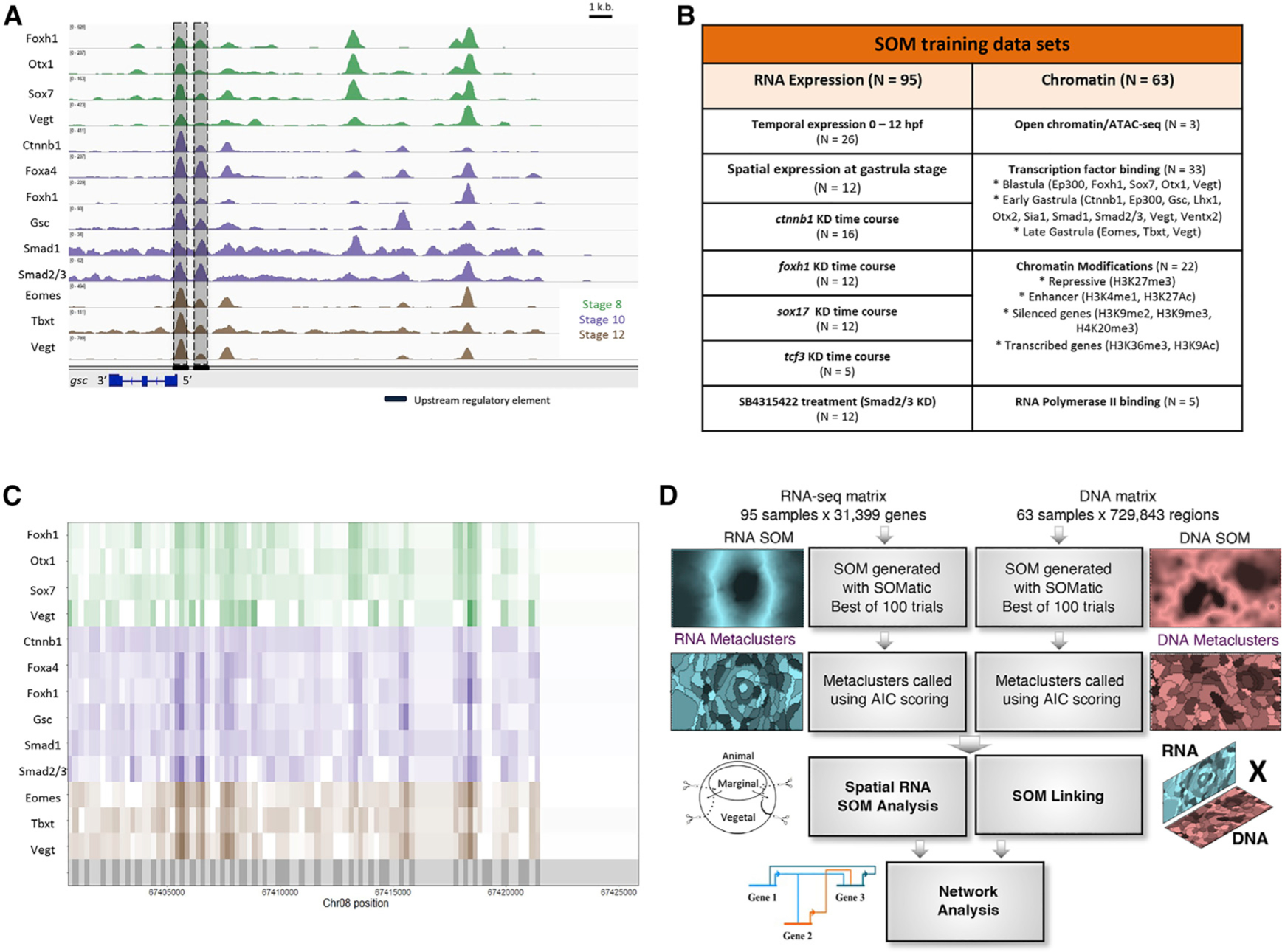
Using self-organizing maps (SOMs) to discover ME GRN (A) Genome browser view of TF binding during *X. tropicalis* development. Shown are maternally expressed (Foxh1, Otx1, Sox7, Vegt, Ctnnb1, Smad1, and Smad2/3) and zygotically expressed (Foxa4, Gsc, Eomes, Tbxt, and Vegt) TF binding in the gsc gene locus. Shaded are the well-characterized proximal, distal, and upstream CRMs, associated with TF binding. Further upstream are binding sites in possibly unexplored CRMs. (B) Datasets used in this analysis, targeting several wild-type and MO-injected embryos at developmental stages important for ME development. (C) The *X. tropicalis* genome is partitioned (grey shadings in bottom track) using ChIP-seq and ATAC-seq peak locations. Each partition is assigned ChIP-seq and ATAC-seq signal quantified as reads per kilobase per million (RPKMs) for all chromatin datasets. (D) The RNA-seq and ChIP-seq/ATAC-seq datasets were each converted into training matrices and clustered using SOM metaclustering using SOMatic. These clusters were then linked using the SOM Linking tool within SOMatic. The pairwise linked metaclusters (LMs) and spatial SOM data were mined for regulatory connections and built into networks.

The scope of the problem of building GRNs with high-throughput genomic data can be illustrated by examining the known regulatory loci surrounding the gene encoding the Spemann organizer TF Gsc (Cho et al., 1991), which contains two regulatory elements near the promoter, called the proximal and distal elements (Watabe et al., 1995), and a farther upstream element (Mochizuki et al., 2000). These DNA regions are bound and controlled by a small set of ME TFs (Koide et al., 2005). However, recent ChIP-seq datasets highlight the possibility of the function of various maternal TFs (Nakamura et al., 2016; Charney et al., 2017b; Paraiso et al., 2019), organizer TFs (Yasuoka et al., 2014), and mesodermal TFs (Gentsch et al., 2013) in regulating *gsc* through these *cis*-regulatory modules (CRMs; Figure 1A). Further, these known elements represent the minority of peaks identified upstream of the *gsc* transcription start site. The chromatin context within this region highlights the need for integrative analysis of genomic datasets.

### Strategy for integration of different highly dimensional genomic data types

To investigate *X. tropicalis* ME gene regulation, we assembled a highly dimensional dataset of 95 RNA-seq and 63 ChIP-seq/ATAC-seq experiments (Figure 1B; Key resources table). The chromatin datasets include the openness of the loci, TF binding, epigenetic modifications, and RNA polymerase II association. The transcriptomic datasets include spatial expression, temporal expression at relevant developmental stages, and knockdown (KD) data of critical TFs and signaling pathways. These data were individually analyzed and collected into two large matrices for unsupervised learning (STAR Methods). For the RNA-seq experiments, gene expression was quantified in transcripts per million (TPM) for each experiment. For the ChIP/ATAC-based experiments, we first partitioned the genome using peak calls identified from these chromatin datasets. Then, we calculated reads per kilobase per million (RPKM) signal for each experiment within each of these partitions. As an example, compare the conversion of the ChIP-seq peaks (Figure 1A) with the RPKM ChIP-seq signals (Figure 1C) in the *gsc* locus. These normalized tracks properly transform the raw signal into a form that our downstream unsupervised neural networks can accept to perform clustering.

Next, we implemented a strategy for highly dimensional data integration through linked SOMs (Jansen et al., 2019) (Figure 1D). This involved performing unsupervised learning through SOMs on each type of data separately, followed by metaclustering to generate a separate RNA SOM and DNA SOM. For the RNA SOM, genes are clustered in terms of similarities in expression: spatially, temporally, and by effects of perturbation. For the DNA SOM, the DNA regions are clustered based on similarities in DNA accessibility, histone modifications, and combinations of TFs bound. These RNA SOM and DNA SOM clusters are then linked such that clustered genes are associated with nearby clustered DNA partitions. Additionally, separately created spatial RNA SOM clusters were incorporated into the network analysis (discussed further below). This combined approach allows for groups of CRMs to be linked to their nearby target gene expression profiles for further network analysis (Figure 1D).

### RNA SOM identifies gene expression modules

To identify different gene expression cohorts present during early *Xenopus* development, we performed unsupervised learning on the RNA-seq experimental data by training a SOM followed by metaclustering (STAR Methods). Due to the experimental matrix being “dominated” by time-course expression data, the trained map displayed a time-course-dependent structure such that genes that have similar temporal profiles, such as *gsc*, *nodal1*, *lhx1*, *osr1*, *hhex*, and *osr2* being located in SOM units in one general area of the 2D map, whereas genes that tend to peak earlier, such as *nodal*, *nodal2*, and *sia1*, were in SOM units in another section (Figure S1A) (Owens et al., 2016). In contrast, although comparisons between gene expression KDs using antisense morpholino oligonucleotides (MOs) and their controls were a minority in this dataset (Figure 1B), they do show local differences on the 2D maps across adjacent metaclusters (Figure S1B). Thus, the metaclustering of the units of the map had the capacity to capture these differences.

**Figure 2.**
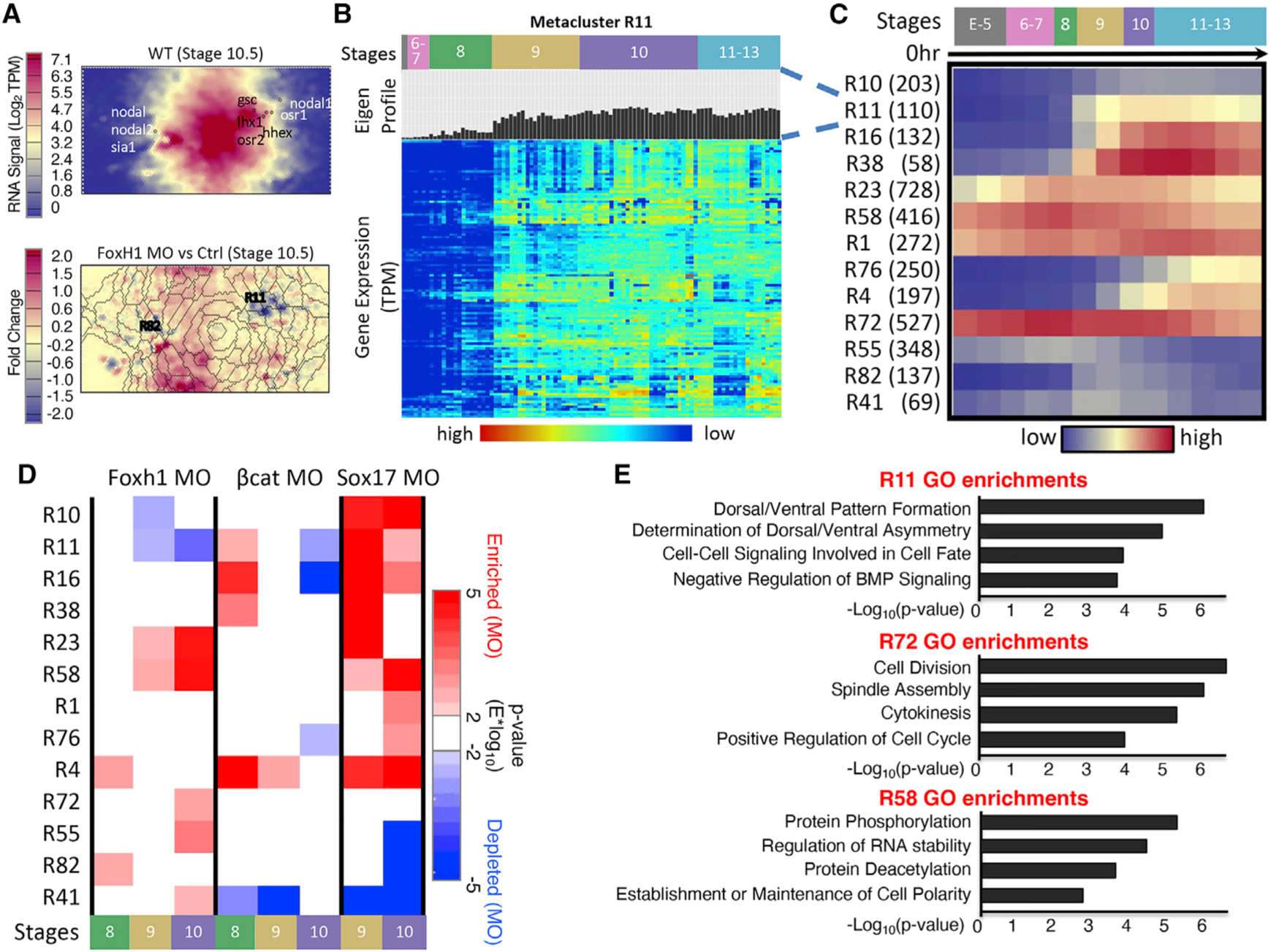
RNA-seq SOM metaclustering reveals developmental gene modules that contain similarly regulated genes (A) SOM slices relating to gene expression signal Wildtype at stage 10.5 and the fold change between Foxh1 MO and control experiments at stage 10.5. Creation of SOM visualization is described in STAR Methods. Metaclusters containing genes from the core ME network show unique temporal dynamics during development. *nodal*, *nodal2*, and *sia* are grouped left and *gsc*, *nodal1*, *lhx*, and *osr2* are grouped right (top). Overlaid metacluster boundaries show the genes that are up- and down-regulated upon Foxh1 MO KD (bottom). (B) Each metacluster is filled with genes with a similar expression profile (labeled “Eigen-Profile”); for example, a heatmap of the genes in metacluster 11 is shown. (C) Heatmap of average temporal expression profiles of genes belonging to 13 RNA metaclusters. Parentheses after RNA metaclusters indicate number of genes in each RNA metacluster. (D) Two-tailed Wilcox hypothesis analysis applied on gene metaclusters. Each metacluster responded to each MO experiment differently at different time points. (E) GO term enrichments for genes within three example RNA SOM metaclusters. Each metacluster had unique functional enrichments supporting the coherence of these clusters. See Figure S1.

In all, we recovered 84 distinct RNA SOM metaclusters that capture the different gene expression profiles present in the included data (Figures 2A and S1B; Table S1A) (labeled R# for each RNA metacluster). Genes that share similar expression profiles across all experiments such as dorsal ME genes activated during midblastula stage, including *nodal*, *nodal2*, and *sia1*, clustered together in metacluster R82. Organizer genes *gsc* and *hhex*, which showed transient zygotic expression peaking at stage 10, clustered together in metacluster R11 (Figure 2B). Meanwhile, genes in metacluster R76 (Figure 2C), which include *foxa2* and *gli3*, did not become highly expressed until mid-gastrula stage 11 and steadily increased until stage 13. In addition to spatiotemporal expression, genes within each metacluster showed distinct responses to perturbation experiments (Figure 2D). For example, the 110 genes in metacluster R11 had similar responses to multiple MO perturbation and temporal conditions. Notably, the genes in this group were down-regulated in stage 10 after inhibiting Foxh1 expression (Foxh1 MO experiment), whereas the 728 genes in R58 and the 527 in R72 were up-regulated. To further show that each metacluster is distinct, we performed Gene Ontology (GO) enrichment analysis on each (Figure 2E). Metacluster R11 contained genes with functions related to dorsal/ventral patterning and cell fate, whereas metacluster R72 had genes related to cell proliferation. See Table S1B for other GO term analysis of metaclusters. The differences in biological functions and properties among these GO term lists suggest that the RNA SOM distinguishes sets of genes based on their expression behaviors under different conditions.

### DNA SOM identifies chromatin states and combinatorial TF binding

**Figure 3.**
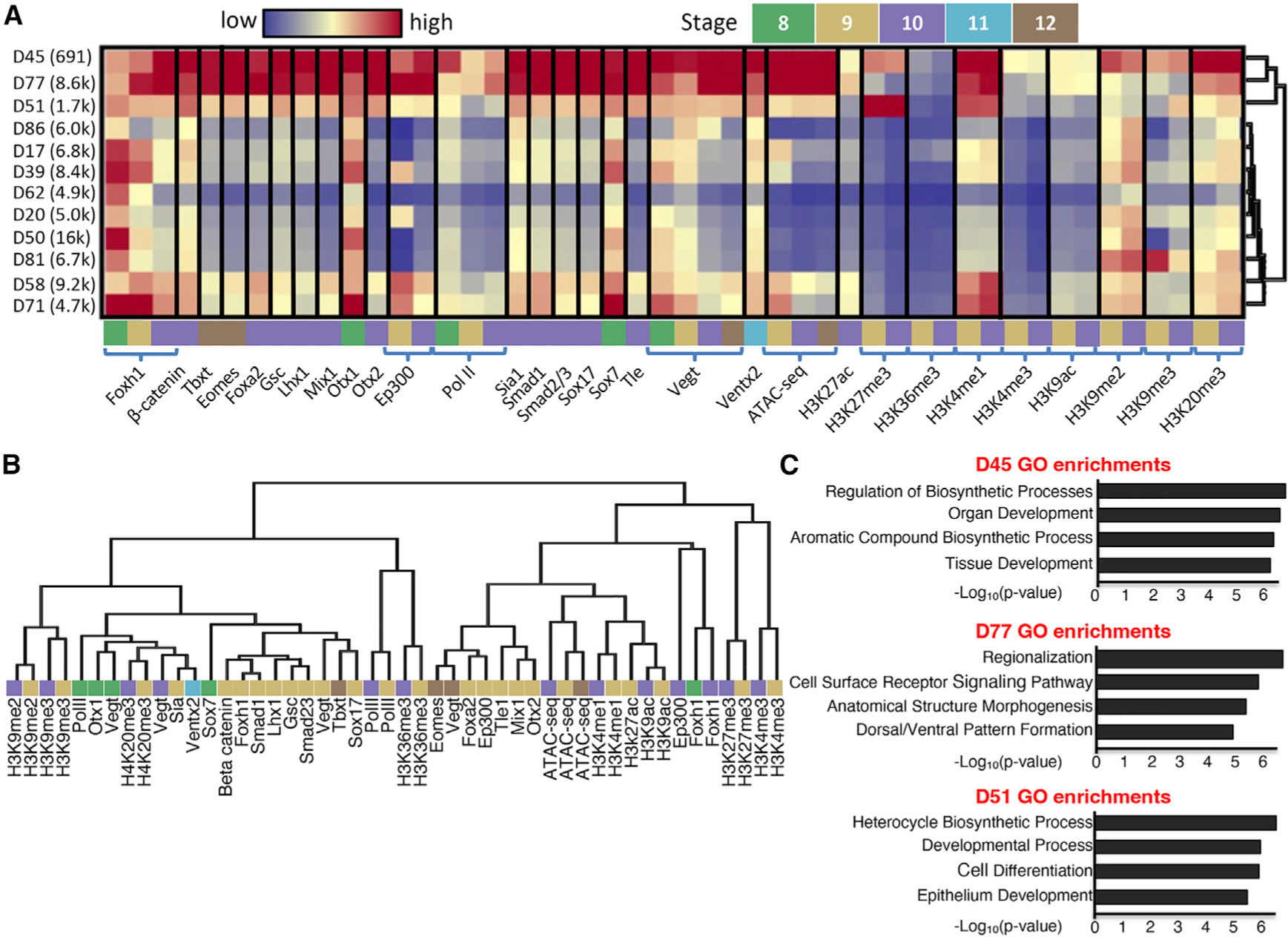
SOM-based clustering shows Foxh1 co-binding and functional gene modules during gastrulation (A) Heatmap of Foxh1 ChIP-enriched metaclusters that visualizes the different patterns of co-regulation present in Foxh1-bound CRMs. The heatmap is initially expressed as TPMs and then maximum normalized. Blue and red represent regions with low and high signals, respectively. (B) Experiment hierarchy of ATAC/ChIP-seq data after metacluster correction. The developmental stages of each experiment are indicated by the same color coding as (A). (C) GO term enrichments for genes nearby genome regions within three example ATAC/ChIP SOM metaclusters.

To prepare the data from the collected ChIP-seq/ATAC-seq experiments for machine learning, we separated the *X. tropicalis* genome into 731,726 genome partitions using called peaks from each experiment (Figures 1C and S3) and computed RPKMs for each experiment over these regions. We then performed unsupervised learning on this matrix with a SOM, and further metaclustering identified 88 distinct DNA profiles present in the data (see Figure 3A for Foxh1-enriched metacluster profiles; see Figure S3 and Table S2A for all metacluster profiles) (labeled D# for each DNA metacluster). Like ChromHMM, these clustered partitions are differentiated by histone marks according to different chromatin states, such as H3K4me1-marked active or primed enhancers (metacluster D71 and D58; Figure 3A) (Heintzman et al., 2007; Creyghton et al., 2010; Buenrostro et al., 2013), H3K9me2/3- and H4K20me3-marked heterochromatic regions (metacluster D72 and D29; Figure S3) (Schotta et al., 2004), and unmodified regions (metacluster D9; Figure S3) (Hontelez et al., 2015). Additionally, the hierarchical clustering over these metacluster profiles (Figures 3B and S3) shows Polycomb repressive H3K27me3 marked regions (Cao et al., 2002) are separated from other chromatin marks in metaclusters D45 and D84 (Figure S3). Similarly, promoter regions marked by H3K4me3 ChIP-seq signals clustered together (D28 and D87; Figure S3) (Santos-Rosa et al., 2002). Interestingly, metacluster D51 has a strong H3K27me3 and H3K4me1 signal, which indicated that this metacluster contains inactive promoters and putative poised enhancers, whereas metacluster D77 replaced the H3K27me3 signal with H3K27ac, which indicated active promoters and Ep300-positive enhancers.

Next, we searched within enhancer-marked regions and visualized interactions of known TF co-bindings via 2D-SOM. For example, previously, we have shown that the maternally expressed endodermal TFs Otx1, Vegt, and Foxh1 can co-bind CRMs, and Otx1 and Vegt synergistically activate endodermal gene expression during cleavage to early blastula stage 8 (Paraiso et al., 2019). In this analysis, Figure S1C highlights that Otx1, Vegt, and Foxh1 ChIP-seq data showed considerable overlap and simultaneous enrichment in metaclusters D77, D71, and D50 (see Figure 3A for their full DNA signal profile). Second, there are significant metacluster overlaps (D20, D39, D58, D71, D77, D45, and D51) between Ep300 and Foxh1 at stage 9 (Figure S1D), indicating a close association between these two factors. Lastly, unlike the early binding of Foxh1 during blastula stages 8–9, Foxh1 binding during early gastrula stage 10 is enriched near dorsal ME genes and is associated with the Nodal co-factor Smad2/3 binding as seen in D77 (Chiu et al., 2014; Charney et al., 2017b). Consistently, Foxh1 binding and Smad2/3 binding were highly correlated as shown by an extensive overlapping heatmap, whereas a heatmap representing Foxh1 binding during blastula stage only partially overlapped with Smad2/3 (Figures S1D and S3). The fact that metaclusters D39, D58, and D71 are free of Smad2/3 but associate with Ep300 indicates that many of the Foxh1-bound regions have Nodal signaling-independent activity. These analyses illustrate the advantage of presenting the ChIP-seq data with a SOM analysis to visually inspect TF-TF interactions and uncovering functional differences of closely related TFs.

Outside known interactions, we find some surprising combinations of TF binding. For example, there is a substantial overlap between the Foxh1 and Gsc SOM maps from the stage 10.5 gastrula (Figure S1D). Interaction between Gsc and Foxh1 has not been well documented, but there is evidence that they directly interact and regulate the expression of the endodermal gene *mix1* (Izzi et al., 2007). Our SOM results suggest that such an interaction may be more widespread during ME specification. Next, the binding of mesodermal regulator Tbxt (Smith et al., 1991) at stage 12 and the endodermal regulator Sox17 (Hudson et al., 1997; Mukherjee et al., 2020) at stage 10.5 correlated well with each other (Figures S1E and S3). This finding indicates that Sox17 and Tbxt bind to similar locations in the genome even if they are expressed in different locations within the embryo. If Sox17 remains bound to these regions until stage 12, this could indicate either a competitive or an independent interaction between Tbxt and Sox17 at stage 12 to generate distinct mesodermal and endodermal lineages. In support of the latter, the expression patterns of Tbxt and Sox17 are also non-overlapping in mice (Lolas et al., 2014). In all, this provides evidence toward a possible conserved mutual exclusion mechanism between Tbxt and Sox17 regulating mesoderm and endoderm development. Lastly, at early gastrula stage 10, the binding of dorsal ME factors Ctnnb1 (β-catenin; Wnt signaling TF) (Nakamura et al., 2016; Heasman et al., 1994) and Foxh1 (Nodal signaling co-factor) (Chen et al., 1996) clusters with the ventral specifying TF Smad1 (BMP signaling TF) (Graff et al., 1996; Afouda et al., 2020) (Figures S1F and S3). Some of these CRMs that show interactions with Wnt, BMP (bone morphogenetic protein), and Nodal signaling pathways may represent the nodes critical in controlling the formation of the dorsal-ventral axis during early embryogenesis. These newly identified combinatorial interactions of TFs underline the usefulness of SOM analysis and would be the topic of further research.

### Distinct genes and consensus DNA binding motif profiles are associated with different DNA SOM metaclusters

To further characterize DNA metaclusters, we performed GO enrichment analysis on the genes whose TSS (transcriptional start site) was the closest to the regions within each DNA metacluster (Figure 3C; see Table S2B for the full list). Embryonic processes correlated with the gene set associated with DNA regions in metacluster D45 are linked to organ and tissue development, while those near metacluster D77 are associated more specifically with morphogenesis and patterning. Additionally, the genes near regions in metacluster D51 are enriched for GO terms associated with cellular and developmental processes. When matched with the RNA metaclusters, these genes were highly enriched in R4, R16, and R76 (see Figure 2A for these profiles), which were all characterized by expression at later time points. The GO analysis thus indicated the genome segments in these DNA metaclusters are used in different transcriptional programs and, thus, require differential gene regulation.

In order to identify the TFs that may control the expression of genes with these distinct metaclusters (D45, D51, and D77), we performed consensus DNA binding motif analysis on each metacluster. After removing the shared motifs among the metaclusters, 63 unique TF motifs to metacluster D45 were recovered, such as Smad2/3, Sox7, and Ventx. These are well-known TFs involved in ME development (Lagna et al., 1996; Ault et al., 1996; Schmidt et al., 1996; Zhang et al., 2005). Metacluster D77 contained 56 unique TF motifs, including Foxa2 and Tcf3 (also known as E2a), which are important in the regionalization of ME (Zorn and Wells, 2009; Wills and Baker, 2015). Finally, 37 TF motifs, including Gata6, which is important for endoderm development (Afouda et al., 2005), are found in metacluster D51. D51 also includes the Tead1 motif, a known repressor in stem cells (Maeda et al., 2002), and the regions in D51 are also decorated with the repressive H3K27me3 mark. Based on these analyses, we concluded that the DNA SOM clustering managed to separate the genome partitions into groups with different biological functions.

### A spatial RNA SOM discovers independent spatiotemporal gene modules

**Figure 4.**
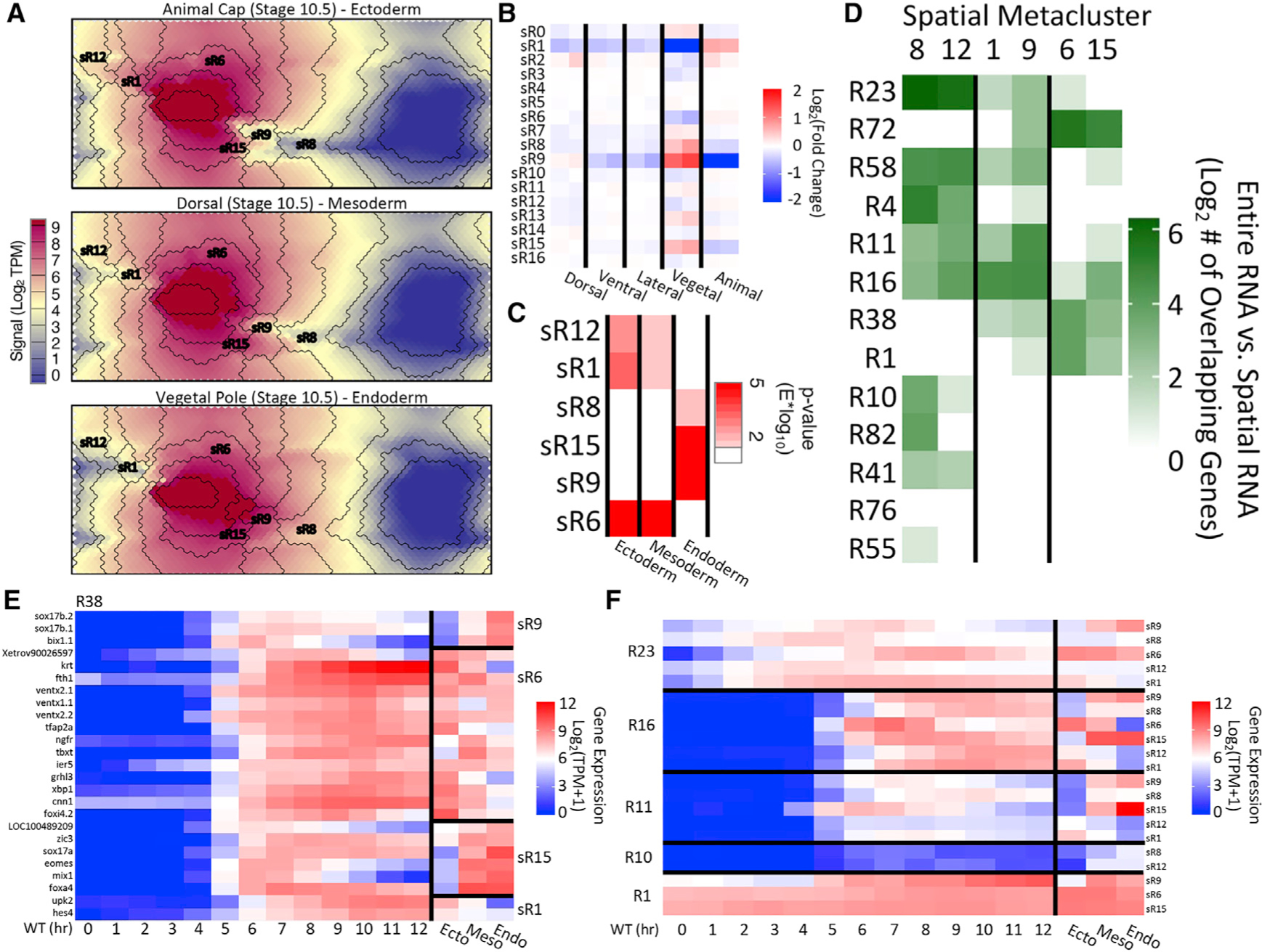
RNA metaclusters can be further segregated by spatial RNA SOM (A) SOM slices from the spatial RNA SOM analysis corresponding to RNAs from the animal, dorsal, and vegetal explants with overlaid spatial RNA metacluster (sR) boundaries. Some important sR locations are noted. (B) Heatmap of the fold change of genes within sRs over whole-embryo signal, indicating enrichment and reduction of genes in particular RNA metaclusters. (C) Heatmap of statistical difference between gene expression in each tissue and the whole embryo. Six sRs showed statistically significant differences in ectoderm/mesoderm or in endoderm. (D) Joint membership of genes in sRs and RNA metaclusters from the full RNA dataset. Rows and columns are hierarchically clustered. (E) Temporal (from wild type) and spatial gene expression profiles for genes in sR9, sR6, sR15, and sR1 and R38. (F) Average temporal and spatial gene expression profiles for genes in R23, R16, R11, R10, or R1, based on sRs.

Unlike the MO KD data, which were successfully incorporated into the SOM metaclustering (Figure 2D), genes in the full RNA SOM did not separate on the map based on their spatial expression profiles. This was due to the full RNA SOM being too focused on the temporal data provided, and so we decided to perform a parallel analysis to provide further insights. For this, we trained a separate SOM, based on just the spatial RNA data from dissected early gastrula (stage 10.5) tissues (Blitz et al., 2017). This analysis provided an excellent separation of genes based on their spatial expression (Figure 4A), and the metaclustering separation followed the differential areas of the map well (labeled sR# for each spatial RNA metacluster). sRs sR9, sR8, and sR1 had quite visible differential spatial gene expression, and sR15 and sR8 showed differential gene expression when the average fold change from each experiment to the whole embryo for each metacluster was plotted (Figure 4B). To show statistical significance of this differential expression, we used the hypothesis tool in SOMatic to find that sR1, 6, 8, 9, 12, and 15 were significantly different from whole-embryo expression levels (Figure 4C). sR8, 15, and 9 were enriched in the endoderm (vegetal pole), and sR12, 1, and 6 were enriched in the mesoderm (marginal zones) and ectoderm (animal cap).

To further explore these sets of genes, we overlapped them (Figure 4D) with the full RNA SOM clustering shown in Figure 2C. Hierarchically clustering the sRs based on gene overlap showed three separate sR groupings, sR8/sR12, sR1/sR9, and sR6/15, that had similar overlap with the full RNA SOM. Interestingly, each group contained one metacluster significantly enriched in the endoderm and the other enriched in the ectodermal and mesodermal experiments (e.g., Figure 4C, compare sR8 and sR12), and each grouping had a specific set of full RNA metaclusters (temporal profiles) with which it overlapped. These observations suggested that there might be sets of potential spatial-specific TFs activated simultaneously in different parts of the embryo that bring about the spatial gene patterns we see in the developing embryo.

To ensure that this observation was not an artifact of the clustering method, we plotted the raw profiles of multiple genes in one full RNA metacluster (R38) and classified those genes by their eventual membership in the differential sRs (Figure 4E). Based on the time-course data, these genes are activated at about 5 h of development, and they each have very different spatial profiles. We also plotted the average profiles of each of the genes in each of the metacluster overlaps (Figure 4F). As expected, the temporal profiles of the genes match in each of the full RNA SOM metaclusters. However, we noted significant spatial differences. This prompted us to explore the regulatory elements near these genes to identify the spatial-specific TFs that are driving this behavior.

### Multi-omic data integration of ChIP/ATAC SOM and spatial RNA SOM provides direction of transcription output

Previously, we developed the linked SOM method specifically to integrate scRNA-seq and scATAC-seq datasets (Jansen et al., 2019). Metaclusters of a scRNA-seq SOM were linked to a scATAC-seq SOM to build sets of genome regions that had similar scATAC-seq profiles near genes with similar scRNA-seq profiles. We determined that this linked SOM approach could be implemented similarly with the spatial *Xenopus* RNA-seq and the abundance of ChIP/ATAC-seq data to uncover the TFs that drive the observed spatial patterns.

**Figure 5.**
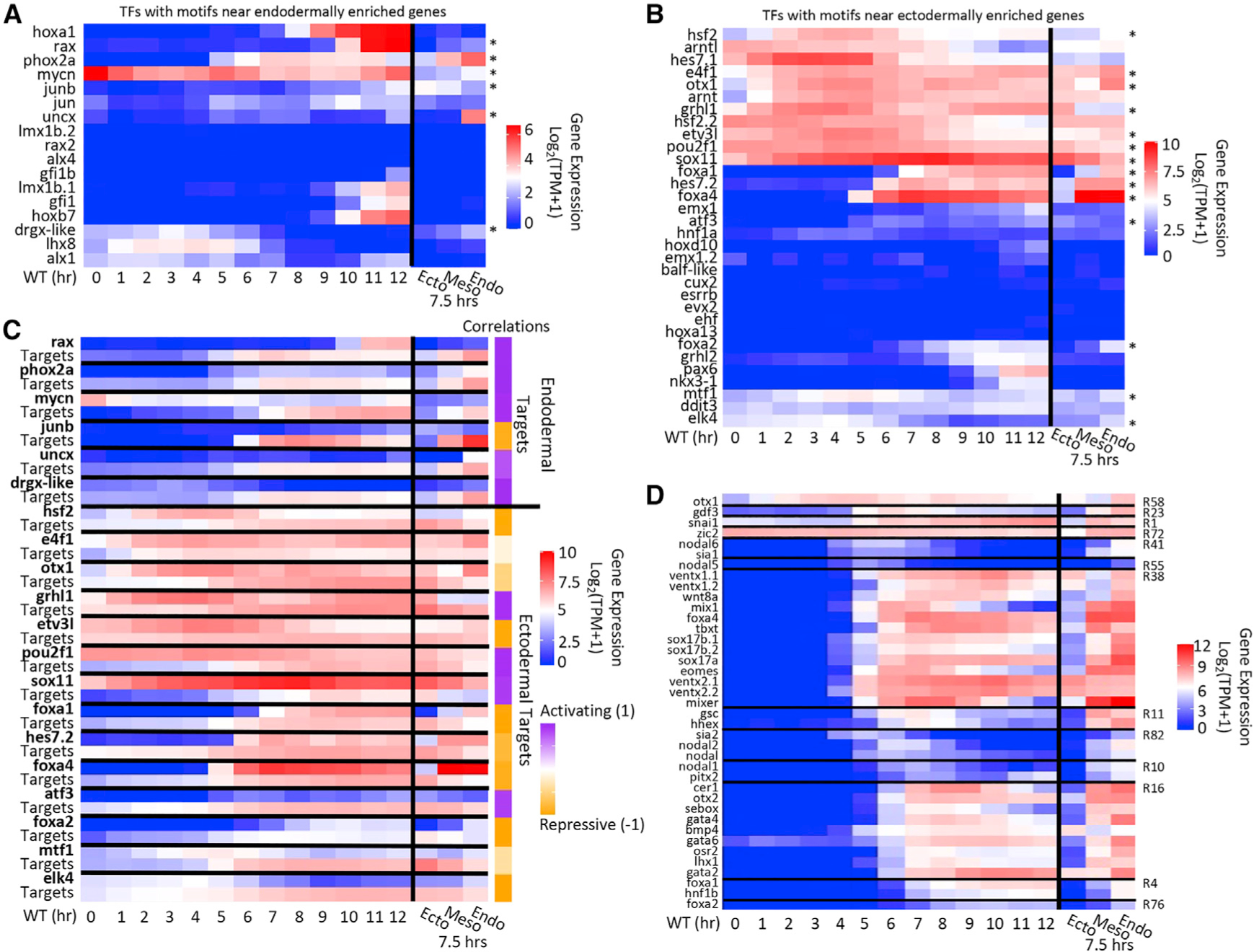
sR assists in identifying candidate TFs for *Xenopus* ME differentiation (A and B) Temporal and spatial gene expression profiles of TFs with motifs found near endodermally (A) or ectodermally (B) enriched genes. Asterisks indicate TFs that show distinct spatial expression. (C) Temporal and spatial gene expression profiles for spatially differential TFs (bold) matched with the average gene expression profile of their predicted targets. Correlations were calculated by comparing their spatial gene expression profiles. (D) The temporal and spatial gene expression profiles of genes important in *Xenopus* ME development, separated by RNA metacluster.

Our goal is to identify specific TF motifs that are enriched among genes that are expressed in specific regions of embryos. We applied the linked SOM approach to the spatial RNA SOM and DNA SOM and generated a linkage between the 16 spatial RNA and the 88 DNA metaclusters, resulting in 1,408 (16 × 88) LMs. A motif search was performed on each LM separately using the human motif database, and motifs that were specifically enriched in a subset of LMs were identified. Of these motifs, we focused on those that appeared near genes in the six differential sRs (Figure 4C) forming three groups: sR8/sR12, sR15/sR6, and sR9/sR1 (Figure 4D). For each pairing, any motifs that appeared in the union of the sRs were filtered out, and motifs that were specific to one metacluster were retained (Figure S4A). To further enrich for TFs with targets showing spatial expression, we searched for the motifs that were shared in at least two of the sets (Figure S4B), and plotted the temporal/spatial expression of 20 candidate TFs that could bind to these motifs (Figures 5A and 5B).

Among those, we selected the TFs that showed a significant (q < 0.05) differential spatial expression. From the motif set near endodermally enriched genes, 6 TFs showed significant differential spatial expression (Figure 5A, asterisks), whereas from the motif set near ectodermally enriched genes, we found 14 TFs (Figure 5B, asterisks). Figure 5C shows the temporal/spatial expression profiles of these TFs alongside the average expression of their predicted targets. By computing the correlation of the spatial signal (and not temporal) from the TFs and their predicted targets, we predicted the overall direction of transcriptional output: potential activating or repressing roles of these TFs. For example, in ectoderm where *foxa1* and *foxa4* expressions are low relative to in endoderm, target gene expression levels are high in ectoderm. This suggests that Foxa1 and Foxa4 had a strong negative correlation between their spatial expression and their potential targets, indicating that they have a role in repressing mesodermal and ectodermal fates.

This plot shows that the majority of predicted spatial regulators of endodermal targets are activators, whereas for the ectodermal targets, most regulators are repressive in nature. This is consistent with the view that ME cells are induced from pluripotent cells that differentiate via an ectodermal default path. For cells to differentiate from an ectodermal to an ME state, certain ectodermally expressed genes need to be reduced in expression (through endodermally/vegetally expressed repressors) and other genes need to be expressed (through endodermally expressed activators). Of the 14 ectodermal TFs, only 4, Ghrl1, Pou2f1, Sox11, and Atf3, had a positive spatial correlation with their targets. Some of these genes are known activators in ectodermal tissues in other organisms (Edgar et al., 2013). Sox11 is a positive regulator of neuronal differentiation in frogs, chick, and mouse (Bergsland et al., 2006; Lin et al., 2010; Chen et al., 2016). Pou2f1 is an activator that is expressed in a wide variety of cell types, including in ectodermal cell lineages in *Xenopus laevis* (Veenstra et al., 1995).

There were 10 TFs with motifs near ectodermally expressed genes that were marked as repressive because their expression was significantly higher in the endoderm. Some of these were already included in the core ME network, such as Foxa1, Foxa2, Foxa4, and Otx1, with others being new potential additions. The data support the notion that these TFs have a repressive role to suppress unwanted ectodermal gene expression in the endoderm. Of the six new TFs, only two are expressed at high enough levels at stage 10 to be considered for being added to the core network: Hsf2 and Hes7.2. Additionally, there are three TFs with motifs that were found near endodermal genes with a high enough gene expression at stage 10 to be considered as well: Phox2a, Mycn, and Uncx. Each of these genes has similar temporal/spatial profiles to the genes from the core ME network (Figure 5D) and were included in the downstream network analysis.

### Generation of a comprehensive ME GRN using multi-omic data integration

In our hand-curated ME GRN (Charney et al., 2017a), a bipartite criterion was used to determine direct TF regulation, whereby a gene was considered a likely TF target if its expression is affected by the perturbation of the TF and if the CRMs near the gene show physical association with the TF. This work required a large investment in manpower and effort, and yet the network was incomplete. With the success of the linked SOM method on finding specific motifs from the spatial RNA and DNA data, we moved to implementing the approach on the full RNA SOM and DNA SOM. This generated a linkage between the 84 RNA metaclusters and the 88 DNA metaclusters, resulting in 7,392 (84 × 88) LMs.

Unlike the spatial/DNA-linked SOM analysis, we were interested in using a set of more specific motifs for known maternal and signaling factors, and as such, we utilized ChIP experiments to build a *Xenopus*-specific DNA binding motif database of Eomes, Foxa2, Foxh1, Gsc, Mix1, Otx1, Otx2, Smad2/3, Sox17, Sox7, and Vegt (Table S3) and motifs for human Tcf7l1/2. When we scanned each LM for these motifs using FIMO with a q of 0.1, we received a set of 271,736 total significant motif instances. Of these, the largest portion belonged to Foxh1 with 134,238 detected motif instances. These initial motif lists were again filtered by LM motif density (STAR Methods) to find significantly (p < 0.05) represented motifs in each LM, which reduced the overall number to 201,157, with 118,722 belonging to Foxh1.

Next, we developed a filtering strategy to focus on the targets active at the developmental time of interest, starting with Foxh1 targets. Limiting the Foxh1 motif instances to those in DNA metaclusters with Foxh1 ChIP signal in the 75th percentile near genes in RNA metaclusters with significant gene expression (>1 TPM) in stages 8–10.5 reduced the number further to 117,253. This small reduction shows that the motif analysis was mostly concordant with the ChIP signal, even before filtering, suggesting that most of the 118,722 genome regions with an identified Foxh1 motif were actually bound by Foxh1. To ensure that we analyzed only active Foxh1 binding sites, we incorporated ChIP-seq/ATAC-seq metaclusters that have an enriched Ep300 signal at stage 9. Application of this filter dramatically dropped the list of potential functional Foxh1 motifs from 118,722 to 26,445 and reduced the number of predicted target genes from 12,831 to 6,717.

To assess the quality of our GRN, we sought to estimate the false positive rate (FPR) for predicted Foxh1 targets. Because a set of true negative gene targets does not exist, we built a list of likely true negative targets for Foxh1 by calling significantly un-changing genes from each of the Foxh1 MO experiments (stages 8, 9, and 10) with DEseq2 (Love et al., 2014) and intersected the lists (Table S4). Of the 5,864 likely true negative target genes, 696 were found within our set of potential targets. This gave the analysis an 11.9% FPR (10.3% FDR), which we deemed acceptable.

**Figure 6.**
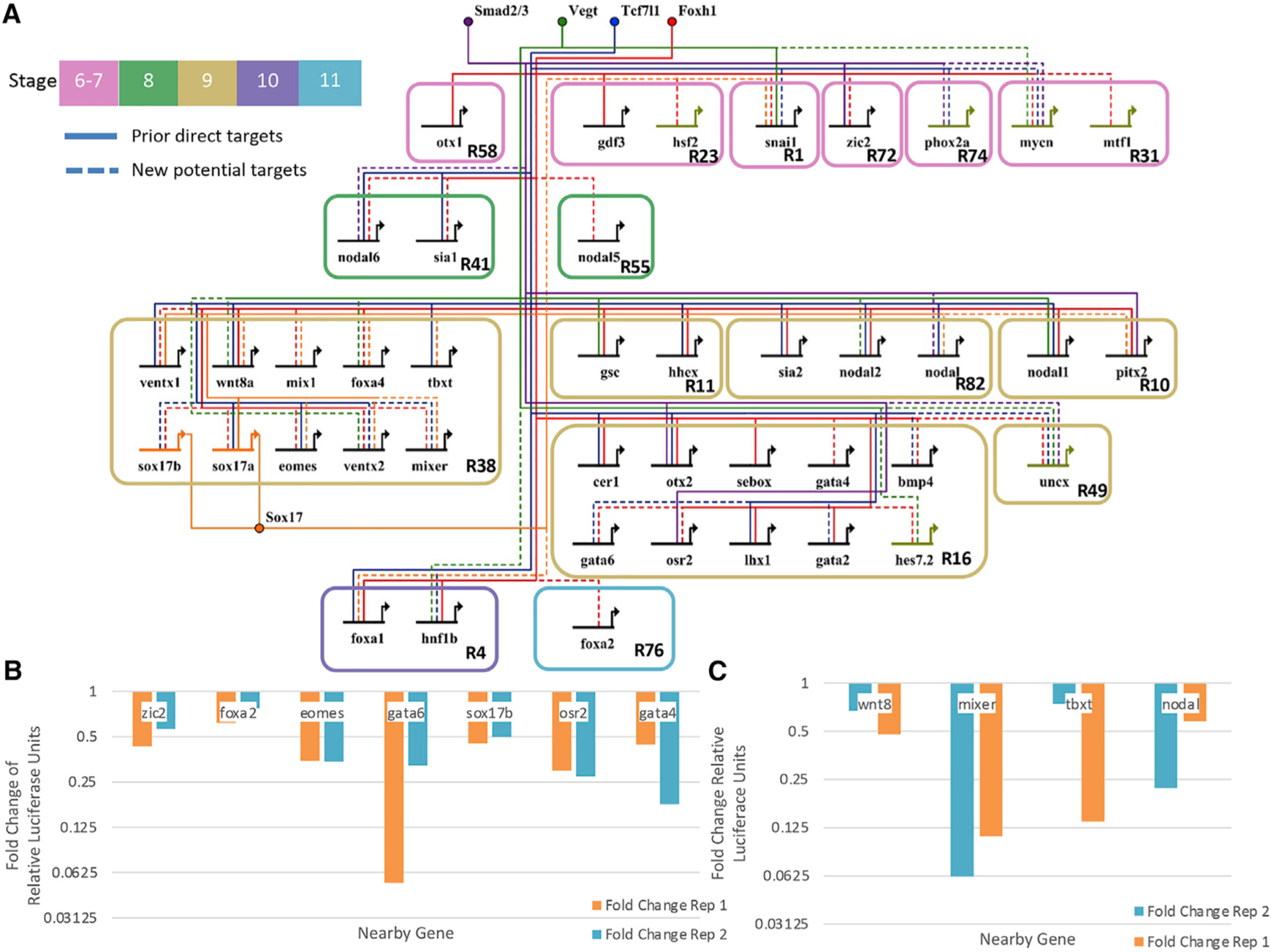
GRN centered on the activity of Tcf7l1, Sox17, Vegt, Smad2/3, and Foxh1 (A) Our predicted developmental GRN. The active CRMs were identified based on the enrichment of their respective TFs, enrichment of Ep300 signal, and DNA binding motif presence. Shown are literature identified targets (“prior direct targets”) and potential new connections (“new potential targets”). Note that only a subset of targets is shown, and the network is focused only on TF and signaling molecule targets. (B) Fold change of relative luciferase units in log scale of putative CRMs comparing Foxh1 binding site mutations over wild type. Each of these shows that enhancer activity depends on Foxh1 binding sites. Two biologically independent experiments were performed. (C) Fold change of relative luciferase units of putative CRMs comparing Sox17 binding site mutations over wild type. Each shows that enhancer activity depends on Sox17 binding sites. Two biologically independent experiments were performed. See Figure S6.

**Figure 7.**
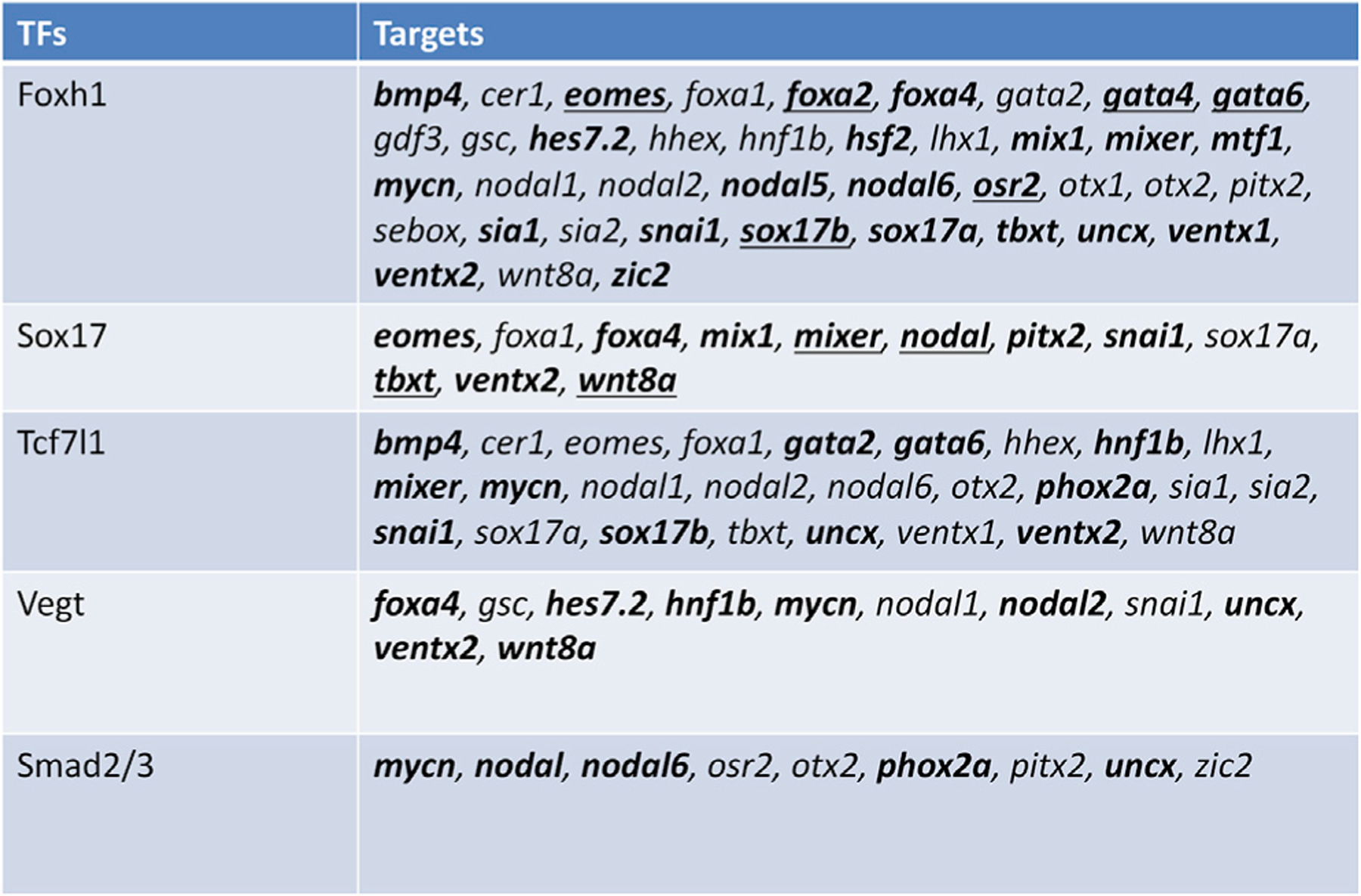
New and known core ME TF targets List of targets in the core ME network for the TFs: Foxh1, Sox17, Tcf7l1, Vegt, and Smad2/3. Bolded entries are new to this analysis. Underlined entries were successfully validated.

To further focus the network, we employed additional constraints by selecting RNA metaclusters that contained genes that regulate gastrulation (Charney et al., 2017a), thereby filtering to 11,295 Foxh1 motifs located near 2,747 unique genes (see Table S5 for full table). Next, we filtered out genes that did not encode TFs or growth factors from our previous works. After this process, 1,492 Foxh1 functional motifs were predicted to be near 242 TFs, and all genes from the curated core ME network (Charney et al., 2017a) remained in the list of 242 (Table S6). This final network includes 2,725 predicted connections for all 12 of our ChIPed TFs with 321 total targets (https://tinyurl.com/3jtrkrct for a full Cytoscape visualization; Figure S5B for full filtering strategy). Finally, we visualized known and predicted network connections of the 36 targets of Foxh1, Sox17, Tcf7l1, Vegt, and Smad2/3 that were present in the core ME network (Charney et al., 2017a) (Figures 6A and 7). Of these, 17 connections for Foxh1, 11 for Sox17, 8 for Tcf7l1, 5 for Vegt, and 2 for Smad2/3 were new to this analysis, which does not include other new connections to the new members of the network. These new potential TF/gene connections inform us what other TFs impinge on ME GRN and thus should improve our understanding of the regulatory processes behind the determination of ME cell states.

To assess whether Foxh1 and Sox17 function through these CRMs, we mutated the DNA sequence motifs that bind these TFs and then compared the activities of the mutant with wild-type reporters (Figures 6B and 6C). In all cases, mutation of Foxh1 and Sox17 binding sites resulted in a decrease in luciferase expression relative to wild-type controls, supporting the notion that these TFs primarily function to activate these genes. We note that wild-type *nodal* reporter’s activity was elevated in response to Sox17 KD, suggesting Sox17 represses *nodal*, whereas the Sox17 binding site mutant’s decrease implicates Sox17 as an activator of this gene. The reason for this discrepancy is currently unclear. We also performed a luciferase reporter analysis of wild-type and Foxh1 and Sox17 MO KD embryos. The MO KD results were similar to that of TF binding mutants (Figure S6D), suggesting that Foxh1 and Sox17 predominantly function as an activator for the genes belonging to metaclusters R38 and R16.

To further test this method, we compared the predicted Tcf7l1 targets with a hold-out Ctnnb1 ChIP dataset (Afouda et al., 2020) (Figure S6D). Of the 26 predicted Tcf7l1 targets, we confirmed 15 during stages 8 and 9, including 6 new Tcf7l1 connections to TFs *sox17b*, *ventx2*, *mixer*, *gata2*, *hnf1b*, and *uncx*. These peak overlaps were significant according to regioneR (Gel et al., 2016) analysis (p = ~3.3 × 10^−3^). Taken together, we conclude that the linked SOM method of regulatory prediction combined with our new filtering methods shows a high-fidelity rate (10 confirmed cases of 12 tested directly for Foxh1 and Sox17; 15/26 confirmed Tcf7l1 regulatory targets from only two stages), while producing significantly more TF-CRM connections than previous methods.

Finally, to determine the potential effect of each of the predicted binding sites above, we examined several different scoring methods and compared those methods’ abilities to predict the effect of each of the 12 validation experiments. Among six methods used (average of each of the following: the H3K4me1; H3K27ac; Ep300; TF ChIP signals to create a TF signal density score; ATAC signals to build a chromatin accessibility score; and a combined score by averaging each of the above scores), the H3K4me1 score performed the best at predicting the downstream effect of validation (Figure S6E; Table S7).

## DISCUSSION

Here, in addition to publicly available *Xenopus* genomic datasets, we generated additional RNA-seq and ChIP-seq data. We then combined three different SOM analyses to prioritize and identify key ME TF targets. This integrated multi-omic approach was successful in accurately recapitulating cellular differentiation programs through network analysis. The generated GRN was validated both experimentally and statistically, to provide a highly confident set of predictions of gene regulation controlling *Xenopus* ME development. These predicted connections included the known core ME networks from previous works (Charney et al., 2017b; Paraiso et al., 2020) and also provided a significant number of new connections. Our analysis represents one of the most data-driven and integrative attempts to recapitulate the GRN of an *in vivo* developmental system.

### Novel network targets of key mesendermal TFs

Numerous genomic analyses of individual TFs have been used to understand early *Xenopus* development (Charney et al., 2017a). In these experiments, combining a single, or a few, ChIP-seq dataset(s) and RNA-seq datasets in wild-type and perturbed states has been used to identify direct transcriptional targets of TFs. A major limitation of this type of analysis is that target identification using a combination of ChIP peaks and large gene expression differences in MO loss-of-function analysis could miss small expression differences. By using an integrative approach that contextualizes a single TF ChIP-seq binding site with the binding of a multitude of regulatory proteins and correlating the binding with the expression of nearby genes, we improve on the previous approaches by leveraging multiple large datasets and receive ~25× as many potential actionable targets for Foxh1 (2,747 in this work compared with 109 in previous works) (Chiu et al., 2014). The usage of multiple types of RNA experimentation in the core analysis was critical to this success because SOMs built on smaller subsets of the data generated less complex clusterings, which led to less specific linked metaclusterings and, thus, fewer actionable targets (~201,000 versus ~102,000 motifs pre-filtering; 2,725 versus 44 connections in the final filtered network).

Of the 40 genes from the core ME network (Charney et al., 2017b), 34 had predicted functional Foxh1 motifs (Figure 7), among which 14 genes were previously confirmed Foxh1 targets. Although some genes, such as *cer1*, *lhx1*, *otx2*, and *sebox*, were previously shown to be regulated directly by Foxh1, *bmp4*, *gata4*, *gata6*, and *osr2* were never implicated as direct Foxh1 targets. Additionally, the metacluster of these genes, R16, also included nine additional predicted targets, such as *hoxd1* and *irx2*, which are critical to axis and pattern formation, respectively. At present, their roles in early ME formation are unknown. Another interesting metacluster is R38, of which only one of the potential Foxh1 targets had previous evidence, *wnt8a*. The majority of core ME genes in R38 (except *tbxt*), including *sox17a* and *sox17b*, which is active in a different region from *wnt8a*, were found to be similarly targeted by Foxh1. Comparing the temporal profiles of R16 and R38 in Figure 2A shows that these clusters have very similar temporal profiles, except genes in R38 being expressed at a higher level than those in R16. This suggests that although Foxh1 regulates the expression of these genes, underlying mechanisms regulating these two metaclusters are different.

The predicted ME network indicated that most of the genes in R38 were regulated by Sox17, whereas none in R16 were predicted. Genes in R38 also maintained a higher gene expression level than those in R16. One speculation is that this difference in gene expression level is due to the positive feedback loop of Sox17 (Sinner et al., 2004; Howard et al., 2007) pulling each of these genes in lockstep with its expression. We tested the model using reporter genes driven by the CRMs of *mixer*, *tbxt*, and *wnt8a* and validated that the output is regulated by both Foxh1 and Sox17 TF input *in vivo* (Figures S5A and 5C). Additionally, the stage 10 expression of genes in metacluster R1 (in particular *snai1*) peaks at nearly the same time point as R38. This is the only maternally and zygotically expressed metacluster with this peak and was the only one predicted to be regulated by Sox17. Based on the current validation experiments, we conclude that many of the newly predicted interactions between TFs and CRMs are likely to have relevant function *in vivo*.

### Enhanceosomes, cooperativity, and antagonism

Although the focus of this work was to elucidate the important CRMs for gene regulation, an important component of the linked SOM analysis, the ATAC/ChIP-seq SOM, revealed interesting clustering of TF binding suggestive of active enhanceosomes. The output of this SOM has shown consistency with known TF-TF interactions, such as that of endodermal maternal TFs (Paraiso et al., 2019), Spemann organizer TFs (Yasuoka et al., 2014), and mesodermal T-box TFs (Gentsch et al., 2013). This unbiased multi-omic clustering approach renders support for the importance of these respective enhanceosomes, complexes of TFs on enhancers. In the future, chromatin clustering with additional data is likely to reveal other interesting enhanceosome biology relevant to development.

Enhanceosomes positively regulate gene expression. The Ep300 co-activator is a histone acetyltransferase, and its interaction with CRMs is one of the frequently used genomic markers of enhancer regions (Heintzman et al., 2007). MEME (Multiple Expectation maximizations for Motif Elicitation) analysis of Ep300 peaks reveals the enrichment of Sox and Fox TF binding motifs, indicating that Ep300 is recruited to DNA via Sox and/or Fox family TFs. Consistent with this observation, we find that early Ep300 binding clusters with Foxh1 (at stage 9) and late Ep300 binding clusters with Foxa2 (at stage 10). Interestingly, Ep300 did not cluster with Sox7 nor Sox17, indicating that other Sox family TFs, such as Sox3, may be responsible for Ep300 recruitment. We also note that Smad2/3 binding, which is a sign of Foxh1-mediated Nodal signaling activity, had a very poor correlation with Ep300 (of the 7,707 Smad2/3 CRMs, only 41 overlapped with a significant Ep300 ChIP signal). This suggests that Ep300 interaction is dynamic. It is initially recruited to the potential sites by maternally expressed Sox and Fox TFs and gradually replaced by other zygotic TFs, such as Foxa2.

Our ATAC/ChIP-seq SOM revealed surprisingly close clustering of ChIP signals for TFs that have distinct spatial expression differences (Figure S3). One of three examples includes the cluster containing dorsally expressed regulator Sia1 (Lemaire et al., 1995) and the ventrally expressed homeobox Ventx2 (Schmidt et al., 1996). This is unexpected because these TFs are known to specify opposing cell types (dorsal versus ventral) and known to be expressed in spatially distinct embryonic regions. One possibility is that these two TFs bind competitively to similar motifs and recruit two distinct enhanceosomes to the same enhancers, depending on the cellular environment. For instance, Sia1 may activate a subset of genes through these enhancers, whereas in a different region of the embryo, Ventx2 may use these same enhancers to repress target genes via recruiting a different combination of co-factors. Alternatively, these enhancers could be similarly regulated in dorsal and ventral regions of the embryo by Sia1 or Ventx2, but other spatial-specific factors could change the topology of the chromatin to target two distinct sets of genes from the same enhancer. Second, we identified this same pattern in other dorsal-ventral pairs of TFs, such as the signaling pathway TF Ctnnb1 (Wnt signaling TF) (Stevens et al., 2017; Heasman et al., 1994), Foxh1 (Nodal signaling co-factor) (Chen et al., 1996), and Smad1 (BMP signaling TF) (Graff et al., 1996). The first two are both important for establishing the dorsal domain of the embryo, while Smad1 helps establish ventral identity. Finally, we note a similar pattern for the TFs Sox17 (Hudson et al., 1997) and Tbxt (Smith et al., 1991), which are critical TFs in forming the endoderm and mesoderm, respectively. A study further focused on these competitive binding locations could help answer how cells dynamically regulate gene expression by sharing similar enhanceosome modules during gastrulation.

In conclusion, we show that linked SOMs are capable of efficiently predicting TF-enhancer interactions to understand the gene regulatory mechanism in an archetypical developmental system. To do this, our approach used a multi-omic dataset to create a highly accurate mechanistic GRN without converting our ChIP/ATAC-seq data into RNA-seq-like data. These results cemented the important role of endodermal TFs, such as Foxh1 and Sox17, in coordinating the expression of many important developmental genes. Our work provides a useful, new platform for the data integration of multi-omic datasets to uncover TF-enhancer interactions in *in vivo* cell and developmental systems. Although we have applied linked SOM for bulk sequencing data, the approach is flexible and can easily integrate other datasets, such as single-cell sequencing datasets.

### Limitations of the study

This work makes predictions of TF binding sites through machine learning and motif discovery and, as such, will have false positives, which we have estimated at ~12%. In addition, *Xenopus* TF motifs are not as well studied as other organisms, and so we were limited in the predictions we could make to those well-studied TFs. Finally, due to practical limitations, we were able to validate targets for only two of our main TFs.

## STAR★METHODS

### RESOURCE AVAILABILITY

#### Lead contact

Further information and requests for resources and reagents should be directed to and will be fulfilled by the lead contact, Ken W.Y. Cho (kwcho@uci.edu).

#### Materials availability

All reporter genes are available upon written request. Antibodies may be available upon written request.

#### Data and code availability

This paper does not report original code. Any additional information required to reanalyze the data reported in this paper is available from the lead contact upon request.

The github for SOMatic was published previously (Jansen et al. al., 2019) and found at: https://github.com/csjansen/SOMatic. Raw and processed RNA-seq and ChIP-seq datasets generated for this study are available at NCBI Gene Expression Omnibus using the accession GEO: GSE161600.

### EXPERIMENTAL MODEL AND SUBJECT DETAILS

Wild type *Xenopus tropicalis,* approximately 3–6 months old males and females were either obtained from NASCO (University of Virginia) or raised in the laboratory and were maintained in accordance to the University of California, Irvine Institutional Animal Care Use Committee (IACUC). 6–36 months old *X. tropicalis* females were injected with 10 units of Chorulon HCG 1–3 nights prior to use, and were injected with 100 units of Chorulon HCG the morning of use. Eggs were collected into a glass dish coated with 0.1% BSA in 1/9x MMR. Sperm suspension obtained from sacrificed males (6–12 months) was used to *in vitro* fertilize the eggs. Ten minutes after fertilization, the embryos were dejellied with 3% cysteine in 1/9x MMR, pH 7.8 and are then ready for further manipulation.

### METHOD DETAILS

#### ChIP-seq and ATAC-seq

Majority of ChIP-seq datasets were obtained from NCBI’s Gene Expression Omnibus (see Key Resources Table). For newly generated datasets, ChIP-seq was performed as previously described (Chiu et al., 2014) at the appropriate developmental stage. The antibodies and conditions for these datasets:

- 30 μg of published Vegt antibody (Sudou et al., 2012) per 2000–3000 embryos
- 30 μg of published Mix1 antibody (Sudou et al., 2012) per 2000–3000 embryos
- 30 μg of published Sia1 antibody (Sudou et al., 2012) per 2000–3000 embryos
- 4 μg of Sox7 rabbit polyclonal peptide antibody (Genscript) per 100 embryos; the peptide antibody was designed against a region in the Sox7 transactivation domain in the C-terminus with the sequence QVSQASDIQPSETS
- 3.5 μg of Ventx2 rabbit polyclonal antibody per 100 embryos; the antibody was generated by Covance, Inc., using a GST fusion to Ventx2.2 amino acids 2–153, upstream of the homeodomain.
- 2.5 μg of Smad1/5/8 antibody (Santa Cruz Biotechnology sc-6031x) per 100 embryos

Libraries were generated using NEXTflex ChIP-seq (Bioo Scientific, Cat# NOVA-5143–01) kit, quality tested using an Agilent Bioanalyzer 2100, quantified using KAPA qPCR, and sequenced using Illumina sequencers at the UC Irvine Genomics High Throughput Facility.

ATAC-seq was generated by Bright et al., 2021.

#### Gene knockdown and RNA-seq

Published RNA-seq datasets for different embryonic tissues and experimental conditions were obtained from NCBI’s Gene Expression Omnibus (see Key Resources Table). For the MO experiments, 2 ng/embryo of *ctnnb1* MO (Mukherjee et al., 2020), 20 ng/embryo *foxh1* MO (Chiu et al., 2014; Charney et al., 2017b), 10 ng/embryo each of two *sox17* MOs (targeting *sox17a* and *sox17b1/2*; Mukherjee et al., 2020) or 4 ng/embryo *tcf7l1* MO (Liu et al., 2005) were used. For the knockdown of receptor-mediated Smad2/3 phosphorylation, embryos were treated with SB4315422 at 100uM as previously described (Chiu et al., 2014; Charney et al., 2017b). For each condition, embryos were harvested at the appropriate developmental stage adhering to the *Xenopus* developmental table (Nieuwkoop and Faber, 1958). RNA samples were collected from embryos using the acid guanidium isothiocyanate method (Chomczynski and Sacchi, 1987). RNA-seq libraries were generated using Smart-seq2 cDNA synthesis followed by tagmentation (Picelli et al., 2014), quality tested using an Agilent Bioanalyzer 2100, quantified using KAPA qPCR, and sequenced using Illumina sequencers at the UC Irvine Genomics High Throughput Facility.

#### Construction of luciferase reporter genes and assay of CRM activity

Minimal gsc promoter (−104gsc) was PCR amplified from −104gsc/Luc (Watabe et al., 1995) and cloned into the promoterless pGL3 basic vector (Promega), which encodes firefly luciferase, between HindIII and NcoI restriction digestion sites. CRMs were PCR amplified from *Xenopus tropicalis* genomic DNA (primers are listed in Key Resources Table) and cloned into the above vector between the BglII and HindIII restriction digestion sites. Mutant CRMs were constructed by inverse PCR according previously published methods (Fisher and Pei, 1997). Oligonucleotides (see Key Resources Table) spanning the motif to be mutated were designed with base substitutions (Foxh1 motifs AATMHACA were changed to AAGMHAAA and Sox17 motifs ACAAWRG were changed to ATAGWRG) and were used in inverse PCR to generate reporter plasmids containing these mutant sequences. All mutations in these plasmids were confirmed by Sanger sequencing. To examine the activity of each CRM’s responsiveness to TF MO knockdowns, 80 pg of luciferase reporter construct and 8 pg of pRL-SV40 (Promega) were co-injected vegetally into 1-cell stage embryos with and without 20 ng of either *foxh1* (Chiu et al., 2014) or *sox17* MO (Mukherjee et al., 2020). Luciferase reporter construct without a CRM served as a negative control. Injected embryos were harvested at stage 10.5 (early gastrula) by homogenizing 5 embryos in 50ul of 5X passive lysis buffer (Promega). 10ul of lysate cleared of cellular debris by microcentriugation were used per assay for luciferase activities according to the manufacturer instruction of Dual-Luciferase Reporter Assay System (Promega). To assess the effects of mutating Foxh1 and Sox17 binding motifs in CRM reporters, 80 pg of either wild-type or mutant reporter was injected vegetally and embryos were harvested at stage 10.5 as described above and assayed for firefly luciferase activity.

### QUANTIFICATION AND STATISTICAL ANALYSIS

#### Chromatin dataset analysis

ATAC-seq and ChIP-seq reads were aligned to the *X. tropicalis* genome v 9.0 (Mitros et al., 2019) obtained from Xenbase (Karimi et al., 2018) using Bowtie 2 v2.2.7 (Langmead and Salzberg, 2012). ATAC-seq and ChIP-seq datasets were peak called relative to their appropriate input DNA controls using MACS2 v.2.0.10 (Zhang et al., 2008) with default options.

#### Self-organizing map (SOM) training, visualization, and metaclustering general procedure

Self-organizing maps are generated by randomly initializing a specified number of artificial neurons on a hexagonal lattice (number of rows and columns is a parameter) to points in the data space. For each timestep, a data point is randomly chosen from the training half of the data matrix (once per computational epoch) and the closest neuron to this point is found (the winning neuron). Then, every neuron on the lattice is moved toward the data point. The distance moved for each neuron depends on the distance on the lattice from the winning neuron, the learning rate (a parameter), and how many timesteps have occurred (this drops as a negative exponential function compared to time) with the winning neuron moving the most.

The resulting positions for these neurons are, then, scored by finding the average distance between each data point in the full data matrix and the closest SOM neuron. To find the final SOM, the SOM training algorithm is run for a number of trials (for each parameter set attempted) and the trial with the best final score is chosen. As metaclustering will follow SOM training, finding the correct number of rows and columns is not necessary as long as there are plenty of elements in the lattice to find all of the data-dense regions of the n-dimensional experiment space. This can be determined by discovering no single-unit metaclusters in the next step.

The final neuron positions can be visualized into a 2D map for each experiment (dimension) in the initial dataset. For instance, see the first SOM slice in Figure 2A, which represents the wildtype gene expression at stage 10.5. The positions of the hexagonal units represent the connections of the neurons on the lattice and the color is the final position of that neuron (aka signal strength) in the experimental dimension. Each unit is a cluster with a number of closest genes or genome regions associated with it that show similar behavior upon perturbations or among different experiments.

Metaclustering is performed by k-means clustering on the final SOM neuron positions such that the growth of each cluster in each step is restricted to only allowing neighboring neurons on the SOM lattice into each cluster (maintaining the SOM’s structure). The metaclustering is attempted for a number of trials for each metacluster number in a given range and the clustering with the best BIC score (reference) is chosen to be the final clustering (and cluster number). This final clustering can be visualized on the SOM maps as an overlay or as a heatmap showing the representative experimental eigen-profile for each metacluster. Metaclusters can also be tested for enrichment or depletion in any given experimental condition.

#### Chromatin segmentation and DNA-SOM analysis

The *Xenopus tropicalis* v9 reference genome was partitioned using the **partition** tool of SOMatic (Jansen et al., 2019) using the MACS2 peak files with a minimum partition size of 200 bp. Then, a RPKM matrix was calculated using the **regionCounts** tool from SOMatic.

The DNA SOM was built using the **buildSite** tool from SOMatic, using a size of 40 × 60, 100 epochs, 100 trials. SOMatic found 88 metaclusters had the best AIC score using 100 trials. GO term enrichments were found using the XenMine gene ontology tool (Reid et al., 2017).

#### RNA-seq dataset analysis

RNA-seq reads were aligned to the *X. tropicalis* genome v 9.0 (Mitros et al., 2019) obtained from Xenbase (Karimi et al., 2018) using RSEM v 1.2.12 (Li and Dewey, 2011) and Bowtie 2 v2.2.7 (Langmed and Salzberg et al., 2012 and Salzberg et al., 2012) to generate gene expression in transcripts per million.

#### RNA-SOM analysis

The RNA SOM was built using the buildSite tool from SOMatic, using a size of 60 × 90, 100 epochs, 100 trials. SOMatic found 84 metaclusters had the best AIC score using 100 trials. Various SOMatic tools were used to create all of the heatmaps, including the statistical enrichment graph, and GO term enrichments were found using the Xenbase GO term tool.

#### Linking of DNA- and RNA-SOM and network analysis

The **Link** tool in SOMatic was used to convolve the 2 SOMs’ metaclusters, using the nearest gene option and limiting the search area to 1Mb. A specific *Xenopus* option (-Xeno) was used because the Xenbase gtf file is a non-standard format.

For the initial ChIP/ATAC-seq SOM, the regions, including repeat regions, in each metacluster were scanned for motifs using the HOCOMOCOv11 human motif database (Kulakovskiy et al., 2018) with FIMO v4.12.0 using a q-value threshold of 0.1. For the further network analysis, each linked metacluster (LM) was scanned with FIMO v4.12.0 (Grant et al., 2011) using a q-value threshold of .1 using motifs calculated from the *Xenopus* ChIP data. The background for both analyses was calculated using the entire *Xenopus tropicalis* v9 reference genome. For each of the 12 calculated TF motifs, the percentage of regions in each LM with that motif was calculated and used to perform one-tailed z-score enrichment with a q-value of 0.05. These significant TF motif locations were mapped to the linked gene.

#### Gene enrichment analysis for unchanging genes throughout time-course

We used DESeq2 v3.11 (Love et al., 2014) to find significantly unchanging genes by using the altHypothesis=”lessAbs” option (qvalue < .05).

## Supplementary Material

1

2

3

4

5

6

7

8

9

## Figures and Tables

**KEY RESOURCES TABLE:** 

REAGENT or RESOURCE	SOURCE	IDENTIFIER

Antibodies		
*X. tropicalis* anti-Ventx2 rabbit polyclonal antibody	Covance; This paper	N/A
*X. troplicalis* anti-Sox7 rabbit peptide polyclonal antibody	Charney et al. (2017b)	N/A
*H. sapiens* anti-Smad1/5/8 rabbit polyclonal antibody	Santa Cruz Biotechnology	Cat#sc-6031x
*X. tropicalis* anti-Mix1 rabbit polyclonal antibody	Sudou et al. (2012)	N/A
*X. tropicalis* anti-Sia1 rabbit polyclonal antibody	Sudou et al. (2012)	N/A
*X. tropicalis* anti-Vegt rabbit polyclonal antibody	Sudou et al. (2012)	N/A

Chemicals, Peptides, and Recombinant Proteins		

Dynabeads Protein G	Life Technologies	Cat#10003D

Critical Commercial Assays		

NEXTflex ChIP-seq kit	Bioo Scientific	Cat#NOVA-5143-01
Superscript II	Life Technologies	Cat#18064014
KAPA HiFi HotStart ReadyMix (2x)	Kapa Biosystems	Cat#KK2601
Agencourt AMPure XP beads	Beckman Coulter	Cat#A63881
Nextera DNA Library Prep Kit	Illumina	Cat#FC-121-1030

Deposited Data		

*X. tropicalis* genome version 9.0	Hellsten et al., 2010; Karimi et al. (2018)	RRID: SCR_003280; URL: http://www.xenbase.org/
*X. tropicalis* Tbxt Stage 12 and 20 ChIP-seq	Gentsch et al. (2013)	GEO: GSE48560
*X. tropicalis* Eomes Stage 12, ChIP-seq	Gentsch et al. (2013)	GEO: GSE48560
*X. tropicalis* Vegt Stage 12, ChIP-seq	Gentsch et al. (2013)	GEO: GSE48560
*X. tropicalis* Foxh1 Stage 10.5, ChIP-seq	Chiu et al. (2014)	GEO: GSE53654
*X. tropicalis* Smad2/3 Stage 10.5, ChIP-seq	Chiu et al. (2014)	GEO: GSE53654
*X. tropicalis* Gsc Stage 10.5, ChIP-seq	Yasuoka et al. (2014)	DRA: DRA000576
*X. tropicalis* Lhx1 Stage 10.5, ChIP-seq	Yasuoka et al. (2014)	DRA: DRA000509
*X. tropicalis* Otx2 Stage 10.5, ChIP-seq	Yasuoka et al. (2014)	DRA: DRA000508
*X. tropicalis* Ep300 Stage 10.5, ChIP-seq	Yasuoka et al. (2014)	DRA: DRA000505
*X. tropicalis* Tle Stage 10.5, ChIP-seq	Yasuoka et al. (2014)	DRA: DRA000506
*X. tropicalis* H3K4me1 Stage 10.5, ChIP-seq	Yasuoka et al. (2014)	DRA: DRA000573
*X. tropicalis* H3K27ac Stage 10.5, ChIP-seq	Yasuoka et al. (2014)	DRA: DRA000574
*X. tropicalis* control and α-amanitin treated embryos H3K27me3 Stage 11, ChIP-seq	Hontelez et al. (2015)	GEO: GSE67974
*X. tropicalis* H3K27me3 Stage 9 and 10.5, ChIP-seq	Hontelez et al. (2015)	GEO: GSE67974
*X. tropicalis* H3K36me3 Stage 9 and 10.5, ChIP-seq	Hontelez et al. (2015)	GEO: GSE67974
*X. tropicalis* control and α-amanitin treated embryos H3K4me3 Stage 11, ChIP-seq	Hontelez et al. (2015)	GEO: GSE67974
*X. tropicalis* H3K4me3 Stage 9 and 10.5, ChIP-seq	Hontelez et al. (2015)	GEO: GSE67974
*X. tropicalis* H3K9ac Stage 9 and 10.5, ChIP-seq	Hontelez et al. (2015)	GEO: GSE67974
*X. tropicalis* H3K9me2 Stage 9 and 10.5, ChIP-seq	Hontelez et al. (2015)	GEO: GSE67974
*X. tropicalis* H3K9me3 Stage 9 and 10.5, ChIP-seq	Hontelez et al. (2015)	GEO: GSE67974
*X. tropicalis* H4K20me3 Stage 9 and 10.5, ChIP-seq	Hontelez et al. (2015)	GEO: GSE67974
*X. tropicalis* control and α-amanitin treated embryos Ep300 Stage 11, ChIP-seq	Hontelez et al. (2015)	GEO: GSE67974
*X. tropicalis* Ep300 Stage 9 and 10.5, ChIP-seq	Hontelez et al. (2015)	GEO: GSE67974
*X. tropicalis* Ctnnb1 Stage 10, ChIP-seq	Nakamura et al. (2016)	GEO: GSE72657
*X. tropicalis* Foxh1 Stage 8 and 9, ChIP-seq	Charney et al. (2017b)	GEO: GSE85273
*X. tropicalis* Foxa2 Stage 10, ChIP-seq	Charney et al. (2017b)	GEO: GSE85273
*X. tropicalis* RNA Pol II Stage 8, 9 and 10.5, ChIP-seq	Charney et al. (2017b)	GEO: GSE85273
*X. tropicalis* Vegt Stage 8, ChIP-seq	Paraiso et al. (2019)	GEO: GSE118024
*X. tropicalis* Otx1 Stage 8, ChIP-seq	Paraiso et al. (2019)	GEO: GSE118024
*X. tropicalis* Sox17 Stage 10.5, ChIP-seq	Mukherjee et al. (2020)	GEO: GSE148726
*X. tropicalis* Mix1 Stage 10.5, ChIP-seq	This Paper	GEO: GSE118024
*X. tropicalis* Sia1 Stage 10, ChIP-seq	This Paper	GEO: GSE118024
*X. tropicalis* Sox7 Stage 8, ChIP-seq	This Paper	GEO: GSE118024
*X. tropicalis* Ventx2 Stage 11, ChIP-seq	This Paper	GEO: GSE118024
*X. tropicalis* Smad1 Stage 11, ChIP-seq	This Paper	GEO: GSE118024
*X. tropicalis* Vegt Stage 9 and 10.5, ChIP-seq	This Paper	GEO: GSE118024
*X. tropicalis* ATAC-seq Stages 9, 10.5 and 12	Bright et al. (2021)	GEO: GSE145619
*X. tropicalis* wild type embryo temporal profiling 0.0–9.5 hpf, RNA-seq	Owens et al. (2016)	GEO: GSE65785
*X. tropicalis* gastrula stage (Stage 10.5) dissected fragments, RNA-seq	Blitz et al. (2017)	GEO: GSE81458
*X. tropicalis* wild type and Foxh1 MO injected embryos Stage 8, 9 and 10.5, RNA-seq	Afouda et al. (2020)	ArrayExpress: E-MTAB-8555
*X. tropicalis* DMSO- and SB431542-treated (Smad2/3 KD) embryos Stage 8, 9 and 10.5, RNA-seq	Afouda et al. (2020)	ArrayExpress: E-MTAB-8555
*X. tropicalis* wild type and Ctnnb1 MO injected embryos Stage 7–12 RNA-seq	Mukherjee et al. (2020)	GEO: GSE148726
*X. tropicalis* wild type, Control MO injected and Sox17 MO injected embryos Stage 9–10 RNA-seq	Mukherjee et al. (2020)	GEO: GSE148726
*X. tropicalis* wild type and Tcf7l1 MO injected embryos Stage 9–10 RNA-seq	This Paper	GEO: GSE118024

Experimental Models: Organisms/Strains		

*X. tropicalis,* out-bred Nigerian	University of Virginia, NASCO	URL:https://www.enasco.com/

Oligonucleotides		

Template switching oligo	Picelli et al. (2014)	N/A
ISPCR primers	Picelli et al. (2014)	N/A
Indexing primers	Buenrostro et al. (2013)	N/A
Foxh1 MO 5′-TCATCCTGAGGCTCCGCCCTCTCTA-3′	GeneTools; Chiu et al. (2014)	N/A
Tcf7l1 MO 5′-CGCCGCTGTTTAGTTGAGGCATGA-3′	GeneTools; Liu et al. (2005)	N/A
Sox17a MO 5′-AGCCACCATCAGGGCTGCTCATGGT-3′	GeneTools; Mukherjee et al. (2020)	N/A
wt zic2 F: ctgtgagtatttacattttacccttgc wt zic2 R: acaatgctacatgctcgg	IDT	N/A
wtfoxa2 F: cagatttcacacagaaaaattaggatc wt foxa2 R: caccattattctttcaaccaccc	IDT	N/A
wt eomes F: tacatctctataagtatgtgtgca wt eomes R: caggataacagagaaggggct	IDT	N/A
wt gata6 F: aacactcatagtttccctttg wt gata6 R: atctcattatgctaaatagacagagg	IDT	N/A
wt sox17b F: ggttagccagcaggtaactg wt sox17b R: aagcaggagaacttgattataataaag	IDT	N/A
wt osr2 F: gtccctgtacaagtaggacatt wt osr2 R: ggaaggcattttaccaaatcctac	IDT	N/A
wt bmp4 F: ggtggtatttccagggttcccttta wt bmp4 R: aagcagcacactgcaacatttg	IDT	N/A
wt gata4 F: agcatggacatgtttaatggact wt gata4 R: ctatttacagctaataccgctcagtg	IDT	N/A
wt wnt8 F: aatgggcagaatatgagaagagt wt wnt8 R: gttcacagtaggaagtgatctaaagc	IDT	N/A
wt mixer F: gggcaaagtcatgagattggt wt mixer R: aagagcattggtactgccg	IDT	N/A
wt tbst F: gcgttcattttgccaccaa wt tbst R: gtggcaatgcagataaatcaact	IDT	N/A
wt nodal F: acactttaaaaggattaatgggatttatct wt nodal R: gcacttggagtgaatagaatgg	IDT	N/A
wt admp F: atatatatatatatactaacagtatatcttgcccaaag wt admp R: aagtaaacttgcaacttaaaaaattaaattttatttc	IDT	N/A
wt map7d3 F: agttttccttccaccaaagaaaa wt map7d3 R: agcttgcctgtatgggat	IDT	N/A
wt pcdh8.2.1 F: aaatctctttcatattcagccgg wt pcdh8.2.1 R: tgagttgttttatgcaatatattttttatagaggc	IDT	N/A
wt pcdh8.2.2 F: acctaaagtcacatcccatcag wt pcdh8.2.2 R: ttgatgacatcaagaaaggtatctaatc	IDT	N/A
wt pcdh8.2.3 F: ggtgcagtgaatggcttattc wt pcdh8.2.3 R: caccttagtgccttcataattgg	IDT	N/A
wt Pdk4 F: agactaaaactgttataagaatttctaatttttaataaatatttg wt pdk4 R: gtaaagttgcactgctttattttacac	IDT	N/A
wt serpinf2 F: agaaatggtgcaccactg wt serpinf2 R: tcaaaatcatgcactgaaggatcaa	IDT	N/A
wt sfrp2 F: aatgagaaaagtgtggtataaga wt sfrp2 R: acactgctactttttaagacagat	IDT	N/A
wt slc12a3.2 F: gaacatatatgtactatgcacttctaacc wt slc12a3.2 R: ttatgctttattcagaaaatattgtaatatttatatgtg	IDT	N/A
wt zic2 F: ctgtgagtatttacattttacccttgc wt zic2 R: acaatgctacatgctcgg	IDT	N/A
mutant foxa2 F: cagatttcacacagaaaaattaggatc mutant foxa2 R: caccattattctttcaaccaccc	IDT	N/A
mutant eomes F: tacatctctataagtatgtgtgca mutant eomes R: caggataacagagaaggggct	IDT	N/A
mutant gata6 F: aacactcatagtttccctttg mutant gata6 R: atctcattatgctaaatagacagagg	IDT	N/A
mutant sox17b F: ggttagccagcaggtaactg mutant sox17b R: aagcaggagaacttgattataataaag	IDT	N/A
mutant osr2 F: gtccctgtacaagtaggacatt mutant osr2 R: ggaaggcattttaccaaatcctac	IDT	N/A
mutant bmp4 F: ggtggtatttccagggttcccttta mutant bmp4 R: aagcagcacactgcaacatttg	IDT	N/A
mutant gata4 F: agcatggacatgtttaatggact mutant gata4 R: ctatttacagctaataccgctcagtg	IDT	N/A
mutant wnt8 F: aatgggcagaatatgagaagagt mutant wnt8 R: gttcacagtaggaagtgatctaaagc	IDT	N/A
mutant mixer F: gggcaaagtcatgagattggt mutant mixer R: aagagcattggtactgccg	IDT	N/A
mutant tbst F: gcgttcattttgccaccaa mutant tbst R: gtggcaatgcagataaatcaact	IDT	N/A
mutant nodal F: acactttaaaaggattaatgggatttatct mutant nodal R: gcacttggagtgaatagaatgg	IDT	N/A

Recombinant DNA		

– 104 *gsc* minimal promoter-pOLuc	Watabe et al. (1995)	N/A
pRL-SV40	Promega	Cat#E2231
zic2 Luc reporter	This Paper	N/A
zic2 mutant Luc reporter	This Paper	N/A
foxa2 Luc reporter	This Paper	N/A
foxa2 mutant Luc reporter	This Paper	N/A
eomes Luc reporter	This Paper	N/A
eomes mutant Luc reporter	This Paper	N/A
gata6 Luc reporter	This Paper	N/A
gata6 mutant Luc reporter	This Paper	N/A
sox17b Luc reporter	This Paper	N/A
sox17b mutant Luc reporter	This Paper	N/A
osr2 Luc reporter	This Paper	N/A
osr2 mutant Luc reporter	This Paper	N/A
gata4 Luc reporter	This Paper	N/A
gata4 mutant Luc reporter	This Paper	N/A
wnt8 Luc reporter	This Paper	N/A
wnt8 mutant Luc reporter	This Paper	N/A
mixer Luc reporter	This Paper	N/A
mixer mutant Luc reporter	This Paper	N/A
tbxt Luc reporter	This Paper	N/A
tbxt mutant Luc reporter	This Paper	N/A
nodal Luc reporter	This Paper	N/A
nodal mutant Luc reporter	This Paper	N/A

Software and Algorithms		

RSEM v.1.2.12	Li and Dewey (2011)	RRID: SCR_013027; URL: http://deweylab.biostat.wisc.edu/rsem/
Bowtie 2 v2.2.7	Langmead and Salzberg (2012)	RRID: SCR_016368; URL: http://bowtie-bio.sourceforge.net/bowtie2/index.shtml
MACS2 v2.0.10	Zhang et al. (2008)	RRID: SCR_013291; URL: https://github.com/taoliu/MACS
DEseq2 v3.11	Love et al. (2014)	RRID: SCR_015687; URL: https://bioconductor.org/packages/release/bioc/html/DESeq2.html
SOMatic	Jansen et al. (2019)	URL:https://github.com/csjansen/SOMatic
FIMO v4.12.0	Grant et al. (2011)	RRID: SCR_001783; URL: http://meme-suite.org/tools/fimo
IGVv2.3.20	Robinson et al. (2011)	RRID: SCR_011793; URL: http://software.broadinstitute.org/software/igv/
Xenmine/Gene Ontology	Reid et al. (2017)	N/A

## Highlights

- Built a pipeline to create GRNs using highly dimensional multi-omic datasets
- Predict new TF-DNA/TF-TF interactions during mesendoderm development
- Generate the genome-scale vertebrate mesendodermal network with high fidelity
- Develop an SOM metaclusters visualization resource for multiple RNA- and ChIP-seq sets
