# Supplementary material for: Uncovering the mesendoderm gene regulatory network through multi-omic data integration
Source: Cell Rep. Author manuscript; Available in PMC 2022 Mar 12. (PMC8917868; doi:10.1016/j.celrep.2022.110364)
Supplement: 1 [file NIHMS1781190-supplement-1.pdf]

Cell Reports, Volume *38*

Supplemental information

Uncovering the mesendoderm gene regulatory network through multi-omic data integration

Camden Jansen, Kitt D. Paraiso, Jeff J. Zhou, Ira L. Blitz, Margaret B. Fish, Rebekah M. Charney, Jin Sun Cho, Yuuri Yasuoka, Norihiro Sudou, Ann Rose Bright, Marcin Wlizla, Gert Jan C. Veenstra, Masanori Taira, Aaron M. Zorn, Ali Mortazavi, and Ken W.Y. Cho

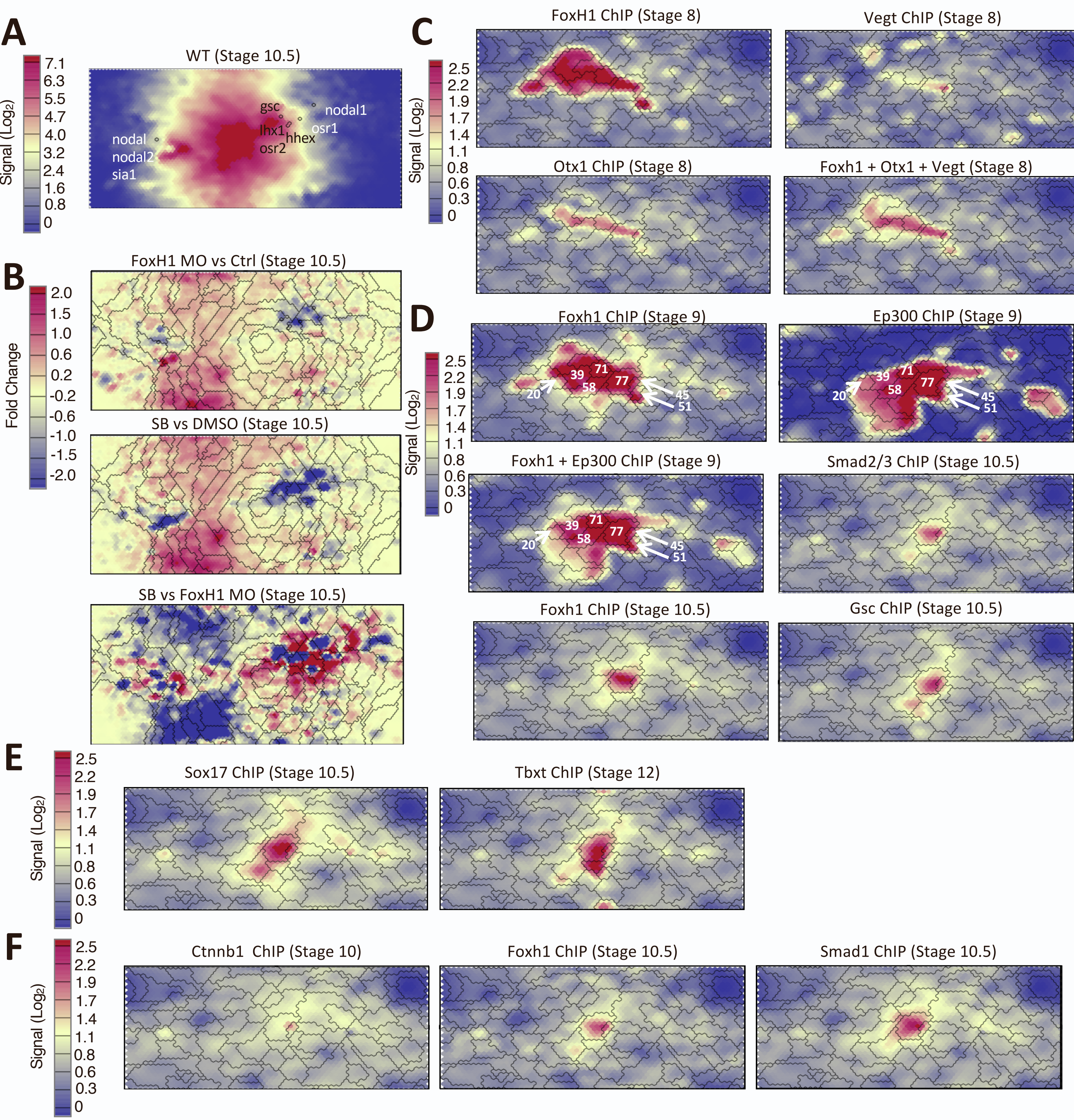

Figure S1. *SOM slices reveal overall structure of the input data.* Related to Figure 2 and 3. **(A)** An RNA SOM slice corresponding to wild-type (stage 10.5). The SOM unit location of various important genes in mesendodermal development are noted. **(B)** RNA SOM difference slices corresponding to the fold change between MO and control experiments. The RNA metacluster divisions are overlaid over the slices. **(C)** DNA SOM slices corresponding to Foxh1, Vegt, and Otx1 ChIPs at stage 8 and one corresponding to their average. The DNA metacluster divisions are overlaid over the slices. **(D)** DNA SOM slices corresponding to Foxh1 and Ep300 ChIPs at stage 9 and their average, followed by DNA SOM slices corresponding to Smad2/3, Foxh1, and Gsc ChIPs at stage 10.5. The DNA metacluster divisions are overlaid over the slices. **(E)** DNA SOM slices corresponding to Sox17 ChIP at stage 10.5 and Tbxt ChIP at stage 12. The DNA metacluster divisions are overlaid over the slices. **(F)** DNA SOM slices corresponding to Ctnnb1, Foxh1, and Smad1 ChIPs at stage 10.5. The DNA metacluster divisions are overlaid over the slices.

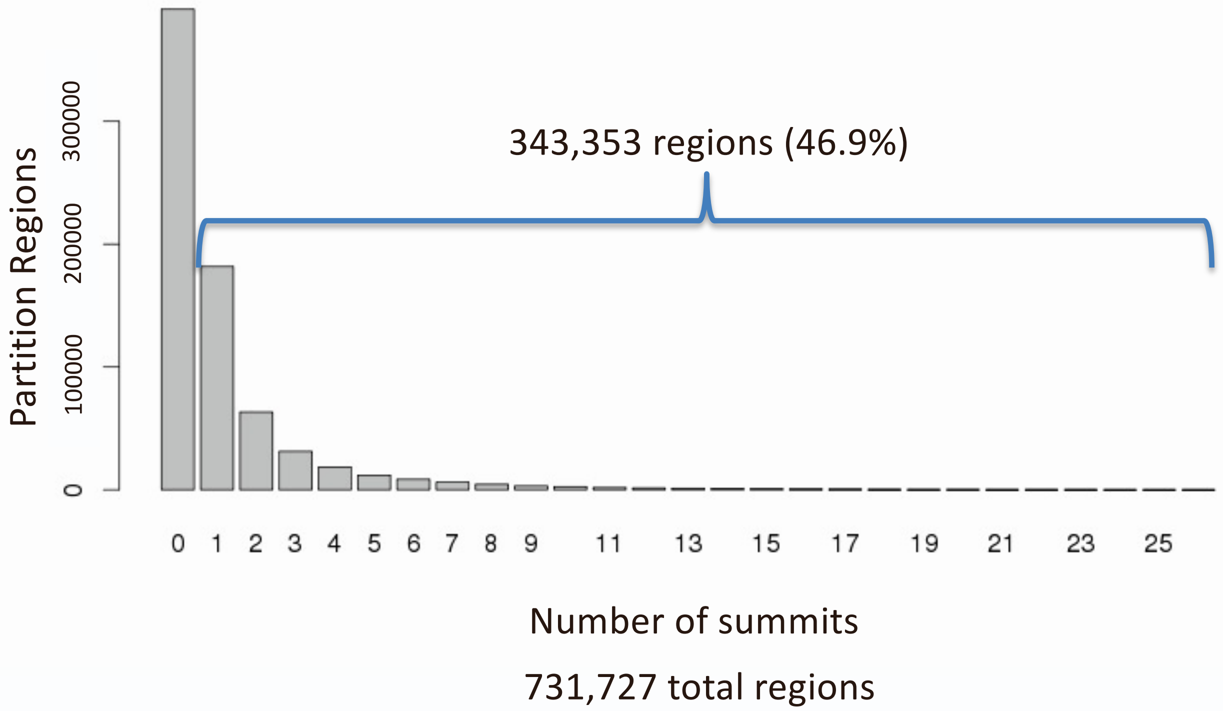

Figure S2. *Distribution of TF ChIP peak summits in final partitioning.* Related to Figure 2. The number of unique TF peak summits within each partition. 46% of partitions contain at least one TF peak summit.

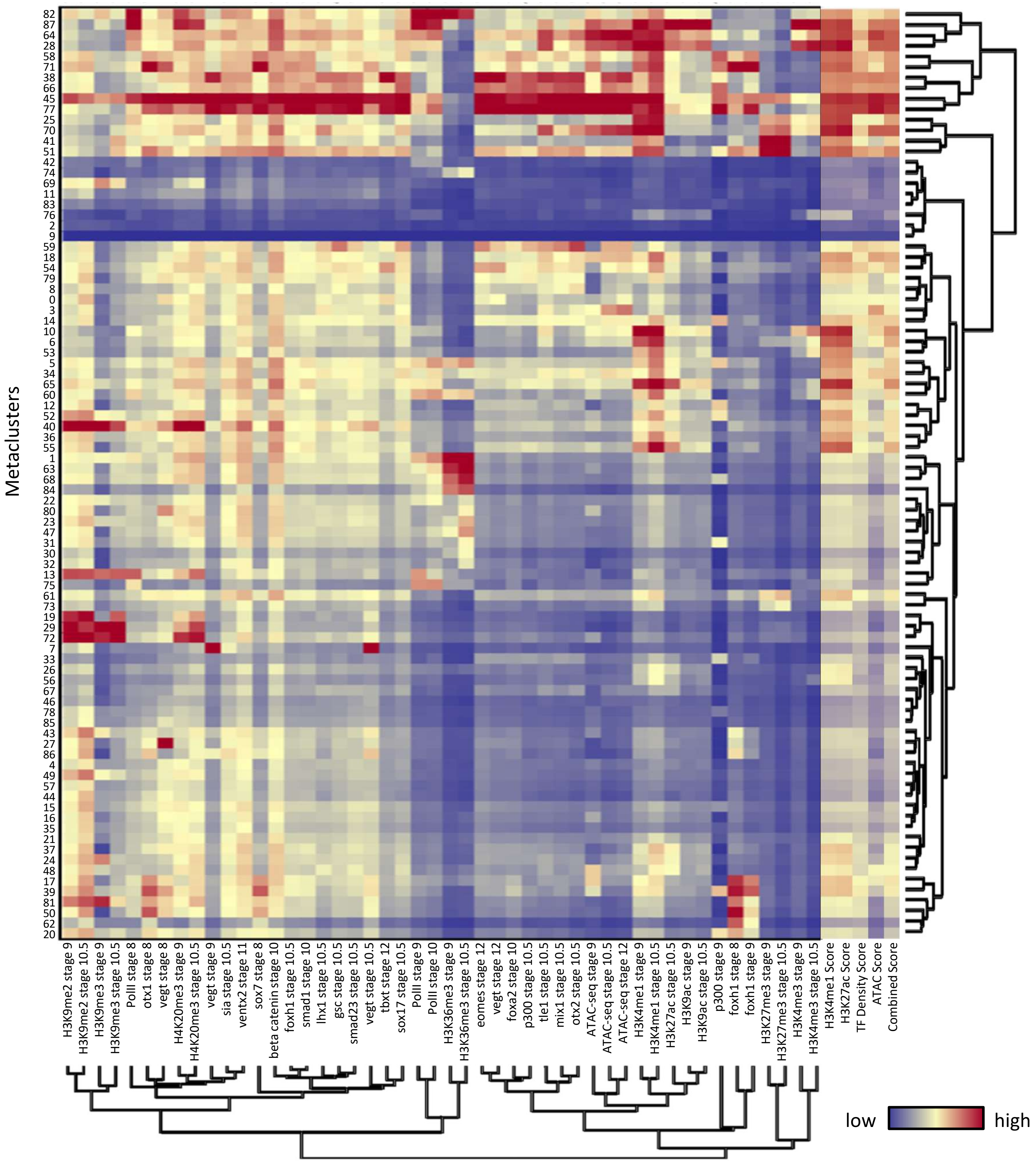
Metaclusters
82
87
64
28
58
71
38
66
45
77
25
70
41
51
42
74
69
11
83
76
2
9
59
18
54
79
8
0
3
14
10
6
53
5
34
65
60
12
52
40
36
55
1
63
68
84
22
80
23
47
31
30
32
13
75
61
73
19
29
72
7
33
26
56
67
46
78
85
43
27
86
4
49
57
44
15
16
35
21
37
24
48
17
39
81
50
62
20
H3K9me2 stage 9
H3K9me2 stage 10.5
H3K9me3 stage 9
H3K9me3 stage 10.5
PolII stage 8
otx1 stage 8
vegt stage 8
H4K20me3 stage 9
H4K20me3 stage 10.5
vegt stage 9
sia stage 10.5
ventx2 stage 11
sox7 stage 8
beta catenin stage 10
foxh1 stage 10.5
smad1 stage 10
lhx1 stage 10.5
gsc stage 10.5
smad23 stage 10.5
vegt stage 10.5
tbxt stage 12
sox17 stage 10.5
PolII stage 9
PolII stage 10
H3K36me3 stage 9
H3K36me3 stage 10.5
eomes stage 12
vegt stage 12
foxa2 stage 10
p300 stage 10.5
tle1 stage 10.5
mix1 stage 10.5
otx2 stage 10.5
ATAC-seq stage 9
ATAC-seq stage 10.5
ATAC-seq stage 12
H3K4me1 stage 9
H3K4me1 stage 10.5
H3k27ac stage 10.5
H3K9ac stage 9
H3K9ac stage 10.5
p300 stage 9
foxh1 stage 8
foxh1 stage 9
H3K27me3 stage 9
H3K27me3 stage 10.5
H3K4me3 stage 9
H3K4me3 stage 10.5
H3K4me1 Score
H3K27ac Score
TF Density Score
ATAC Score
Combined Score
low
high

Figure S3. *Full DNA metacluster heatmap captures known co-regulatory interactions.* Related to Figure 2 and 3. The full set of eigenprofiles revealed that several experiments had very similar results on the collected genomic region clusters. Some of these are known co-regulatory interactions in *Xenopus* or vertebrates in general

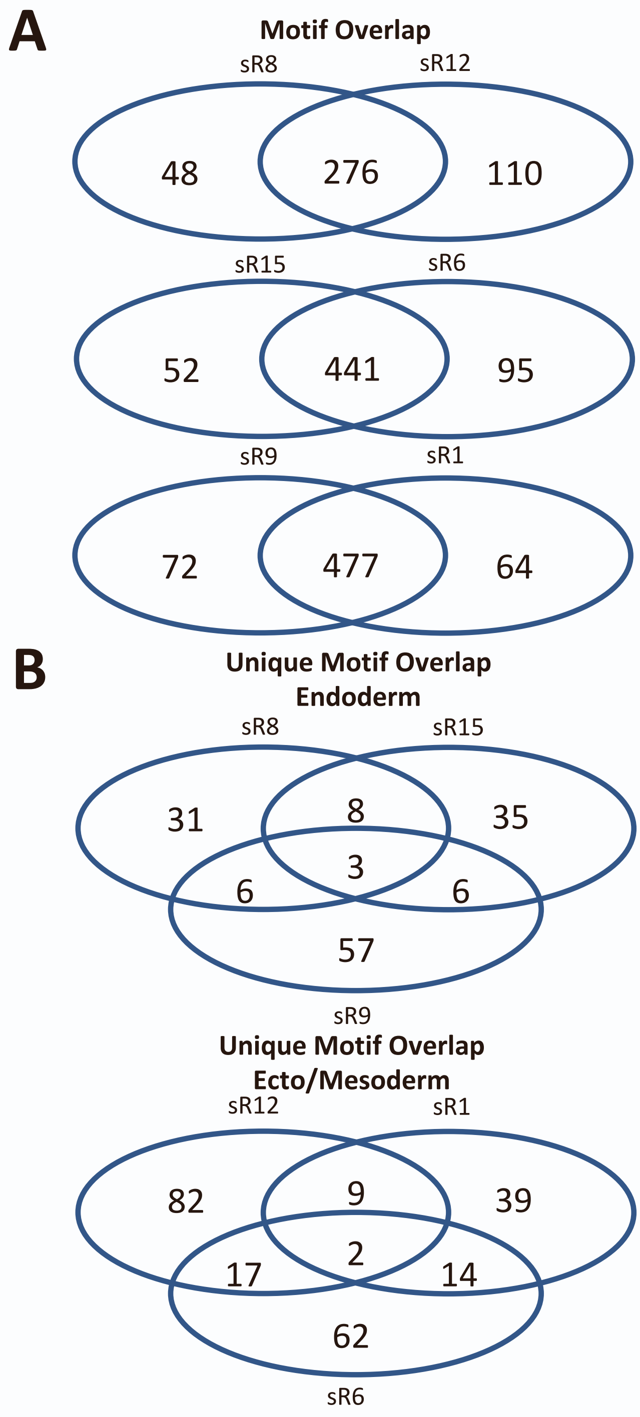

Figure S4. *Motif analysis on linked spatial RNA and DNA SOM metaclusters finds TFs with spatially specific regulation.* Related to Figure 4. **(A)** Venn diagrams showing the motif overlap between spatial metaclusters with similar temporal gene expression profiles. **(B)** The overlaps between motifs found uniquely on each side of the embryo in (A).

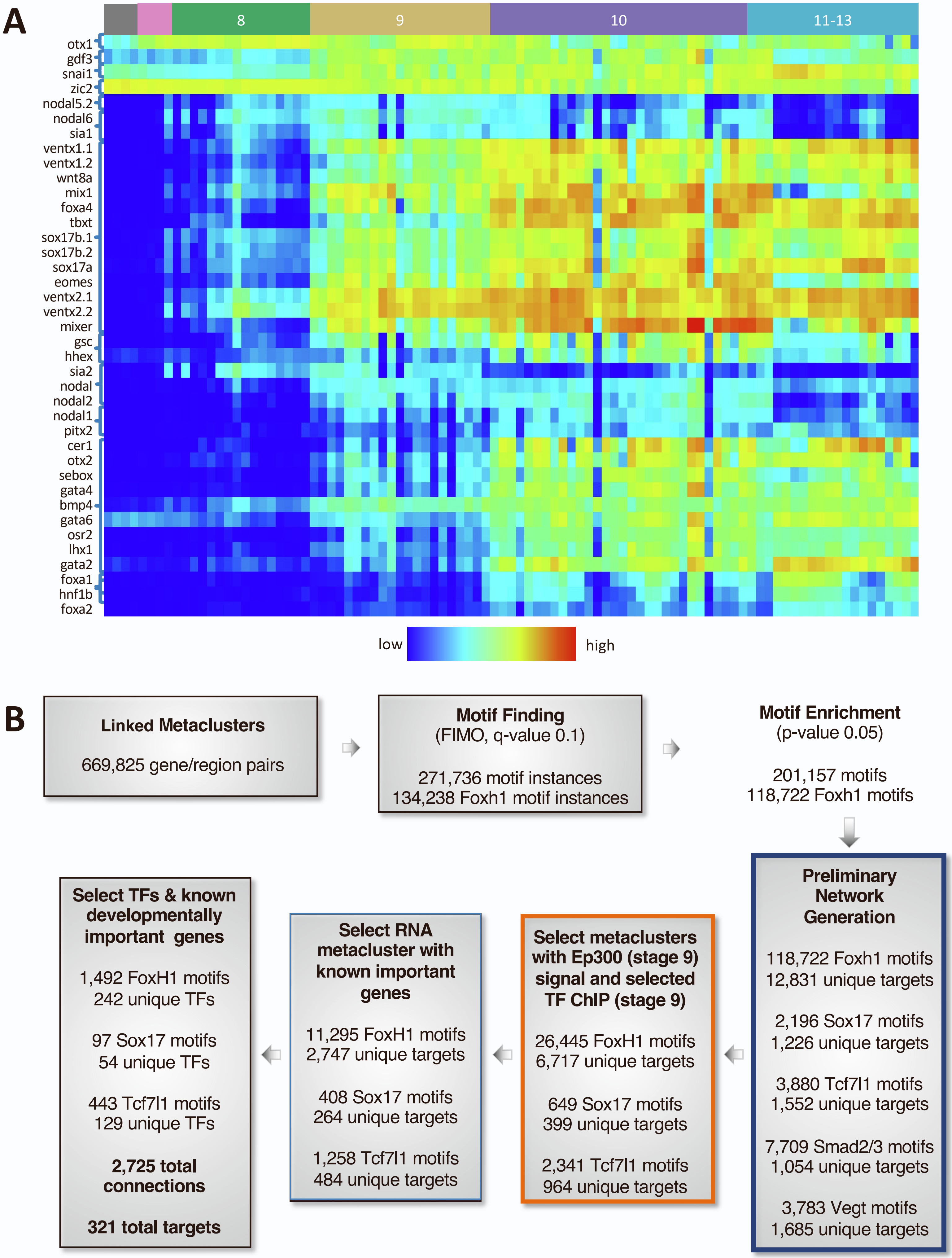
A
8
9
10
11-13
otx1
gdf3
snai1
zic2
nodal5.2
nodal6
sia1
ventx1.1
ventx1.2
wnt8a
mix1
foxa4
tbxt
sox17b.1
sox17b.2
sox17a
eomes
ventx2.1
ventx2.2
mixer
gsc
hhex
sia2
nodal
nodal2
nodal1
pitx2
cer1
otx2
sebox
gata4
bmp4
gata6
osr2
lhx1
gata2
foxa1
hnf1b
foxa2
low
high
B
Linked Metaclusters
669,825 gene/region pairs
Motif Finding
(FIMO, q-value 0.1)
271,736 motif instances
134,238 Foxh1 motif instances
Motif Enrichment
(p-value 0.05)
201,157 motifs
118,722 Foxh1 motifs
Preliminary Network Generation
118,722 Foxh1 motifs
12,831 unique targets
2,196 Sox17 motifs
1,226 unique targets
3,880 Tcf7l1 motifs
1,552 unique targets
7,709 Smad2/3 motifs
1,054 unique targets
3,783 Vegt motifs
1,685 unique targets
Select metaclusters with Ep300 (stage 9) signal and selected TF ChIP (stage 9)
26,445 FoxH1 motifs
6,717 unique targets
649 Sox17 motifs
399 unique targets
2,341 Tcf7l1 motifs
964 unique targets
Select RNA metacluster with known important genes
11,295 FoxH1 motifs
2,747 unique targets
408 Sox17 motifs
264 unique targets
1,258 Tcf7l1 motifs
484 unique targets
Select TFs & known developmentally important genes
1,492 FoxH1 motifs
242 unique TFs
97 Sox17 motifs
54 unique TFs
443 Tcf7l1 motifs
129 unique TFs
2,725 total connections
321 total targets

Figure S5. *Gene expression profiles of genes from core mesedoderm network and network filtering strategy*. Related to Figure 4. **(A)** Heatmap of gene expression for all experiements for each gene from the core mesendoderm network. Columns were sorted by developmental stage of each experiment. Rows were clustered by SOM metacluster and ordered by the starting stage of gene expression for that metacluster. Signal for each experiment was normalized (vertically). **(B)** Motif filtering strategy of network analysis. After using FIMO to find motifs and Motif Enrichment to filter out motifs that are dispersed evenly across the linked metaclusters, the network connections were further filtered by a joint Ep300 and TF ChIP signal separately for each of the TFs tested. Then, we selected RNA metaclusters that contained TFs known to be important for mesendodermal development. Finally, we connected TFs to genes from the core mesendodermal network and known TFs to make the final network.

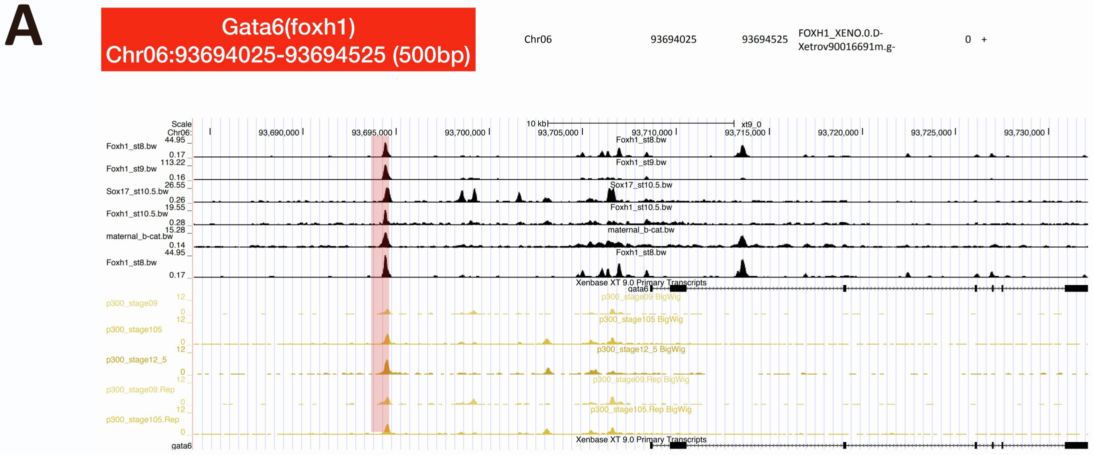

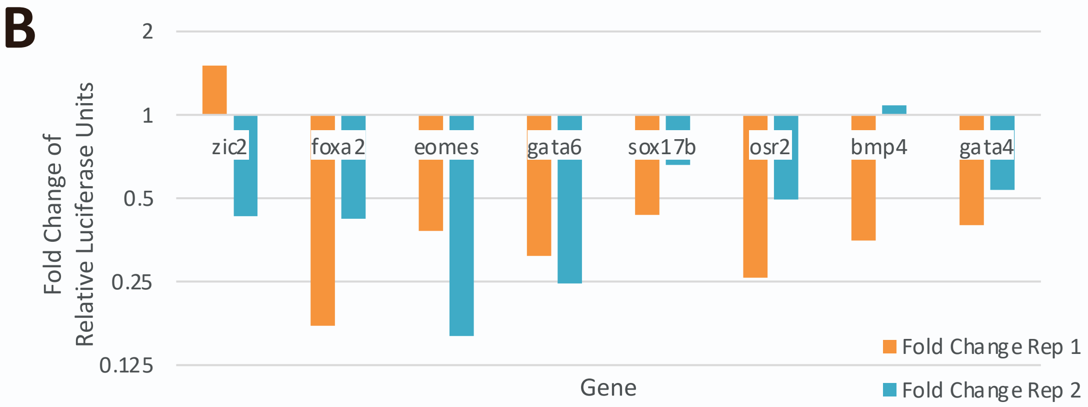

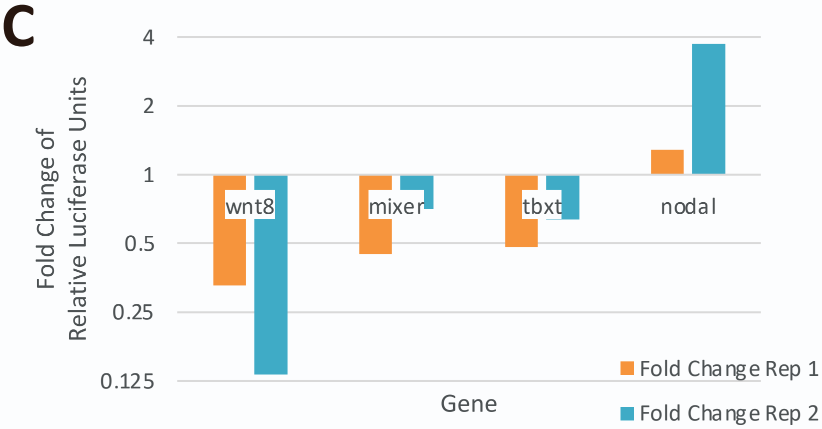

D

Gene	Peak st7	Peak st10.5
bmp4	No	No
cer1	No	No
eomes	**Yes**	**Yes**
foxa1	No	No
gata2	**Yes** (new link)	**Yes**
gata6	No	No
hhex	**Yes**	No
hnf1b	**Yes** (new link)	**Yes**
lhx1	No	No
mixer	**Yes** (new link)	**Yes**
mycn	No	**Yes**
nodal1	No	No
nodal2	**Yes**	No
nodal6	**Yes**	No
otx2	No	No
phox2a	No	No
sia1	**Yes**	No
sia2	**Yes**	No
snai1	No	**Yes**
sox17a	No	**Yes**
sox17b	**Yes** (new link)	**Yes**
tbxt (t)	**Yes**	**Yes**
uncx	**Yes** (new link)	**Yes**
ventx1	**Yes**	**Yes**
ventx2	**Yes** (new link)	**Yes**
wnt8a	No	**Yes**

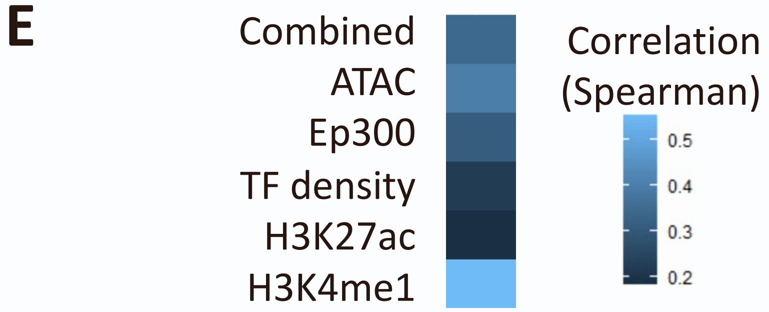

Figure S6. *Final network luciferase validation experiments and importance correlations.* Related to Figure 6. **(A)** A genome browser view of the predicted and tested Foxh1 target element near *gata6* with Foxh1, Sox17, Ctnnb1, and Ep300 ChIP-seq signals for stages 8 - 12. **(B, C)** Fold change of relative luciferase units in Log scale after foxh1 or sox17 MO injection. Reporter gene was injected into the vegetal (mesendoderm) region of embryos with or without co-injected. Each microinjected CRM reporter showed expression in the mesendoderm (Table S9, Figure S6B, C), which suggested that each probed region is a functional enhancer. All reporter gene activities were downregulated in the absence of Foxh1 or Sox17, except a *nodal* enhancer reporter. At present it is unclear whether the genes belonging to R82 are negatively regulated by Sox17 or whether this is due to an isolated action of this *nodal* CRM. **(D)** Tcf7l1 targets and their overlap with a Ctnnb1 ChIP holdout dataset. **(E)** Heatmap of the correlation between each importance score and the effect on each of the 12 validation experiments. H3K4me1 scored the best.
